# Tuning molecular emission of organic emitters from fluorescence to phosphorescence through push-pull electronic effects

**DOI:** 10.1038/s41467-020-16412-4

**Published:** 2020-05-26

**Authors:** Hai-Tao Feng, Jiajie Zeng, Ping-An Yin, Xue-Dong Wang, Qian Peng, Zujin Zhao, Jacky W. Y. Lam, Ben Zhong Tang

**Affiliations:** 1 Baoji AIE Research Center, Shaanxi Key Laboratory of Phytochemistry, College of Chemistry and Chemical Engineering, Baoji University of Arts and Sciences, 721013 Baoji, China; 2 Department of Chemistry, Hong Kong Branch of Chinese National Engineering Research Center for Tissue Restoration and Reconstruction, Institute for Advanced Study, The Hong Kong University of Science and Technology, Clear Water Bay, 999077 Kowloon, Hong Kong China; 3 Center for Aggregation-Induced Emission, SCUT-HKUST Joint Research Laboratory, State Key Laboratory of Luminescent Materials and Devices, South China University of Technology, 510640 Guangzhou, China; 4 Institute of Functional Nano and Soft Materials (FUNSOM), Jiangsu Key Laboratory for Carbon-Based Functional Materials and Devices, Soochow University, 215123 Suzhou, China; 5 Key Laboratory of Organic Solids, Beijing National Laboratory for Molecular Science, Institute of Chemistry, Chinese Academy of Sciences, 100190 Beijing, China

**Keywords:** Electronic materials, Lasers, LEDs and light sources, Displays

## Abstract

Organic emitters with persistent phosphorescence have shown potential application in optoelectronic devices. However, rational design and phosphorescence tuning are still challenging. Here, a series of metal-free luminophores without heavy atoms and carbonyl groups from commercial/lab-synthesized carbazole and benzene were synthesized to realize tunable molecular emission from fluorescence to phosphorescence by simply substituent variation. All the molecules emit blue fluorescence in both solution and solid state. Upon removal of excitation source, the fluorinated luminophores show obvious phosphorescence. The lab-synthesized carbazole based molecules exhibit a huge lifetime difference to the commercially purchased ones due to the existence of isomer in the latter samples. The small energy gap between singlet and triplet state and low reorganization energy help enhance intersystem crossing to contribute to a more competitive radiative process from triplet to ground state. Blue and white organic light-emitting devices are fabricated by using fluorinated luminophore as emitting layer.

## Abstract (web summary)

Though organic emitters with room temperature phosphorescence (RTP) are attractive for various applications, realizing highly efficient and long lifetime emission remains a challenge. Here, the authors report the role of push-pull electronic effects on emission for organic RTP emitters.

## Introduction

Illumination consumes approximately 20% of the civilian electric energy every year, and 40% of that amount is consumed by inefficient incandescent lamps^1,2^. Facing the ever-increasing energy crisis, scientists have made efforts to develop high-efficiency lighting pattern, such as light-emitting devices (LEDs)^3–5^. In LEDs, the emitter plays a vital role in devices^6–8^. However, the internal quantum efficiency (IQE) of LEDs based on fluorescent materials can only reach up to 25% according to the quantum spin theory statistics (the population of singlet excitons to that of triplet excitons is 1:3). Thus, most of the electric energy is wasted and the development of more efficient emitters is thus of great importance in energy conservation. Recently, an IQE of 100% can be theoretically realized by thermally activated delayed fluorescence (TADF) and room-temperature phosphorescence (RTP) emitters through harvesting both the triplet and singlet excitons^9,10^. Up to now, many efficient TADF materials have been reported^11–15^. For phosphorescent emitters, research on their molecular design and emission mechanism is still in its infancy because phosphorescent materials documented so far often contain transition metal atoms^16^, such as iridium and platinum, to overcome the forbidden transition of triplet excitons by virtue of strong spin-orbit coupling (SOC). Unfortunately, due to their high cost, toxicity and instability, metal complex-based phosphorescent materials often are in limited variety. In contrast, pure organic RTP emitters without these drawbacks are attracting increasing interest in the field of optoelectronic devices^17^ chemosensors^18^, bioimaging^19^ and data encrypt^20,21^. Most reported organic RTP molecules contain heavy atoms, carbonyl groups or sulfone groups to import strong SOC effect to promote intersystem crossing (ISC) and radiative transition probability of the lowest triplet state (T_1_) to the ground state (S_0_). Normally, such radiative transition cannot compete with ultrafast non-radiative decay process and phosphorescence is thus seldom observed in organic luminophores at room temperature. How to facilitate ISC and improve radiative relaxation from T_1_ to S_0_ is therefore the key factor to develop highly efficient and ultralong organic RTP molecules.

RTP is not only influenced by molecular vibration and oxygen quenching but also determined by other factors, such as molecular packing and intermolecular interactions. So far, attempts have been made to promote ISC and suppress non-radiative decay, including host–guest interactions^22–25^, H-aggregation^26^, heavy-atom effect^27,28^, crystallization-induced RTP^29–31^, intermolecular electronic coupling^32^, molecular packing^33^, polymer system^34–37^ and so on. Tang also proposed a strategy and showed that the narrow energy gap between the lowest singlet and triplet states (Δ*E*_ST_) and pure π π* configuration of T_1_ are beneficial to achieve efficient and ultralong RTP materials^38^. Generally, crystalline packing is regarded as a prerequisite to increase the radiative transition of triplet state to ground state by rigidifying molecular conformation and vibration. For example, Li and co-workers found that the compact face-to-face packing in crystals helps increase the lifetime and emission efficiency^39^. Although literature review states that ketone or halogen atoms (Br or I) can enhance SOC and the likelihood of intersystem crossing, these functional groups are also apt to increase electronic transition rates of S_*n*_–T_*n*_ and T_*n*_–S_0_ simultaneously. Thus, it is a common phenomenon that increasing phosphorescence efficiency is always accompanied by decreased lifetime in RTP emitters. A feasible strategy to address this issue is to develop an effective RTP system without ketone and halogen atoms.

Previous studies focus mainly on the photophysical properties and structures of RTP emitters, and pay little attention to how push-pull electronic effect affects their fluorescence and phosphorescence emission. Here, we design and synthesize a series of pure organic luminophores by integration of commercial (Cm)/lab-synthesized (Lab)carbazole (Cz), and benzene (namely TCz-F, TCz-H, and TCz-OH). Through tuning their substituent groups on the phenyl ring from hydroxyl to hydrogen, then to fluoro atoms, we can realize control of molecular emission from fluorescence to persistent phosphorescence. In contrast to RTP emitters from lab-synthesized Cz, TCz-F, and TCz-H from commercial Cz (TCz-F-Cm and TCz-H-Cm) show much redder luminescence and longer phosphorescence lifetime (727 ms and 128 ms) in the crystalline state. These values are comparable to those RTP materials reported so far (Supplementary Fig. 1). Through high performance liquid chromatography (HPLC) analysis, such huge difference in lifetime is attributed to an isomer of commercial carbazole, which can dramatically improve RTP property. While TCz-OH-Cm shows only blue fluorescence in both solution and crystalline state with a lifetime of 11 ns, suggesting that molecular structure tuning is mainly responsible for their different photophysical behavior. Moreover, the microrod-like crystals of TCz-F-Cm display good optical waveguide properties. When the size of crystals increases to millimeter, the optical-guiding performance becomes inferior due to serious light scattering. Using TCz-F-Cm as emitter or guest material, blue and white organic light-emitting devices are fabricated.

## Results

### Synthesis

**Fig. 1:**
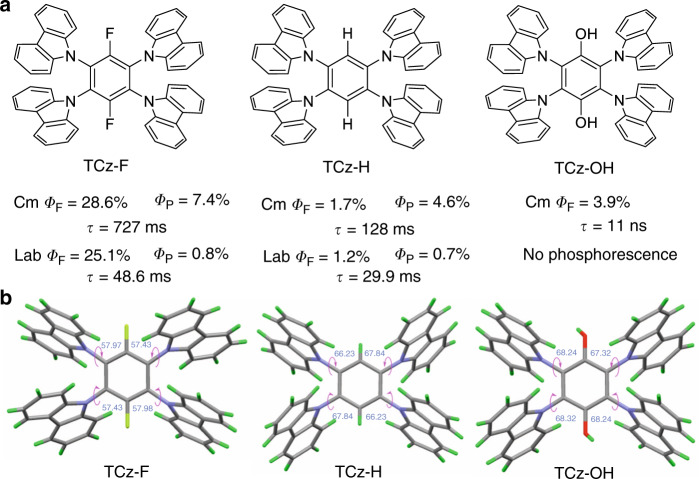
Molecular structures of carbazole-functionalized benzenes. **a** Molecular structures and photophysical properties. **b** Crystal structures of TCz-F, TCz-H, and TCz-OH.

Recently, Liu reported that an isomer (1H-Benz[f]indole) of commercial Cz can dramatically influence RTP of Cz and its derivatives^40^. In order to exclude the effect of isomer, we synthesized carbazole from N-(2-Bromophenyl) aniline according to previous literature^41^ and the purity was confirmed by Waters 1525 HPLC (water-methanol ratio is 30/70) (Supplementary Fig. 2). To evaluate the effect of isomer, we synthesized TCz-F, TCz-H, and TCz-OH through S_N_Ar reaction of commercial/lab-synthesized carbazole with dibromotetrafluorobenzene or hexafluorobenzene followed by necessary reaction in good yields of over 70%. The detailed synthetic route was depicted in Supplementary Fig. 3. Their structures and purity were fully characterized by NMR spectroscopy, high-resolution mass spectrometry (HRMS), HPLC and single-crystal X-ray diffraction (Fig. 1, Supplementary Figs. 4–13 and Supplementary Table 1). Since electron-accepting (A) ability of fluoro group, electrons in TCz-F are apt to transfer from carbazole to difluorophenyl ring. When changing the substituent to electron-donating (D) group (–OH), the resultant molecule (TCz-OH) shows large difference in photophysical properties as compared with TCz-F and TCz-H. From their crystal structures we can see (Fig. 1), all the molecules adopt twisted conformations due to huge steric hindrance of neighboring carbazole units. The dihedral angles between the phenyl core and the carbazole unit fall in the range of 57.43° to 68.32°. It is notable that TCz-F shows better conjugation than TCz-H and TCz-OH due to its less distorted structure. On the other hand, these twisted structures will prevent emission self-quenching in the crystalline state due to strong intermolecular interaction. The thermal stability of TCz-F, TCz-H, and TCz-OH are determined by thermogravimetric analysis. As shown in Supplementary Fig. 14, all the molecules are thermally stable and show high thermal-decomposition temperatures of 432 °C, 429 °C, and 393 °C, respectively.

### Photophysical properties

**Fig. 2:**
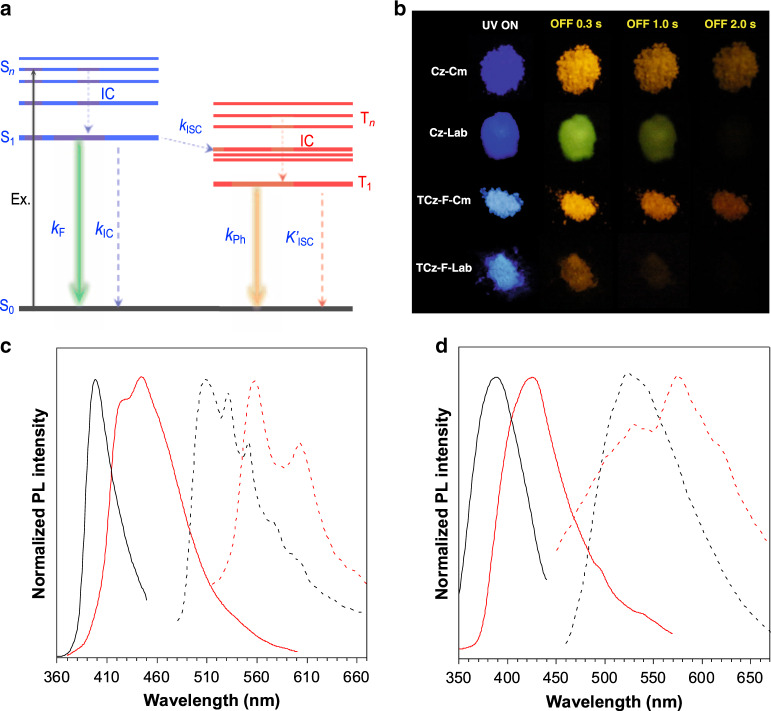
The luminescent images, prompt and delayed PL spectra. **a** Schematic Jablonski diagram for organic emitter. **b** Photographs of Cz-Cm, Cz-Lab, TCz-F-Cm, and TCz-F-Lab taken before and after switching off excitation source. The prompt (solid line) and delayed (dash line) PL spectra of **c** TCz-F-Cm (red line), TCz-F-Lab (black line), and **d** TCz-H-Cm (red line), TCz-H-Lab (black line) in the solid state.

Interestingly, the photoluminescence (PL) of lab-synthesized carbazole (Cz-Lab) shows a hypochromatic shift as compared to commercially purchased carbazole (Cz-Cm). The Cz-Lab emits no RTP in amorphous state. After recrystallization, obvious yellowish-green phosphorescence is observed at room temperature, which is also blue-shifted in contrast to yellow RTP of Cz-Cm (Fig. 2). The UV-vis spectra of TCz-F-Cm, TCz-H-Cm, and TCz-OH-Cm in THF show main absorption peaks at ~ 300 nm and 335 nm with a shoulder peak at around 320 nm due to π–π* transition and intramolecular charge transfer (Supplementary Fig. 15a). The band gaps were calculated from the UV-vis absorption edge to be 3.73 eV, 3.66 eV, and 3.71 eV for TCz-F-Cm, TCz-H-Cm, and TCz-OH-Cm, respectively. Photoexcitation of their THF solutions at 330 nm gives blue fluorescence at 420 nm, 405 nm and 390 nm with a quantum yield of 16.3%, 9.0% and 13.5%, respectively (Supplementary Fig. 15b). However, upon gradual increasing water fraction to 60% in THF solution, PL intensity of TCz-F-Cm decreases accompanied by a red-shift from 423 nm to 438 nm due to the twisted intramolecular charge transfer (TICT) effect (Supplementary Fig. 16). A prerequisite to obtain dark TICT state depends on a D-A molecular structure and twisted conformation in the excited state. During this process, the energy gap between excited state and ground state is decreased to afford red-shifted emission. In the meantime, the dark state of TICT relaxes to the ground state mainly through nonradiative transition, resulting in weak or no emission. Afterwards, the PL intensity increases and reaches up to the maximum at 80% water fraction. However, due to lack of D-A units in TCz-H-Cm, the intramolecular charge transfer is weakened and there is no obvious wavelength change in THF and water mixtures with different water fractions. For TCz-OH-Cm, it also shows obvious TICT effect in THF and water solution. The time-resolved PL spectra of these emitters were also collected in THF and water mixtures (Supplementary Table 2). All the lifetimes fall in the nanosecond range, demonstrating fluorescence nature of the light emission. The crystalline powders of TCz-F-Cm as suggested by powder X-ray diffraction (Supplementary Fig. 17) show red-shifted emission by 20 nm than that in the solution state. Similar phenomena are also observed in TCz-H-Cm and TCz-OH-Cm and the maximum emission peaks locate at 425 nm and 410 nm. The fluorescence quantum yields (QYs) of these crystals are 28.6%, 1.7%, and 3.9%, respectively. Compared with those Cz-Cm based emitters, the lab-synthesized TCz-F and TCz-H (namely TCz-F-Lab and TCz-H-Lab) show much bluer emission in both the solution and solid state (Supplementary Fig. 18). The solid-state fluorescence QYs of TCz-F-Lab and TCz-H-Lab were measured to be 25.1% and 1.2%, indicating the isomer of Cz shows minor influence on their fluorescence efficiency.

In principle, radiative decay from the lowest triplet state to the ground state gives phosphorescence (Fig. 2a). The crystalline powder of TCz-F-Cm emits blue fluorescence upon irradiation by 365 nm UV lamp (Fig. 2b). After removal of the light source for 0.3 s, a brightly yellow afterglow is observed. Even after 2.0 s, the yellow emission is still discernible, indicating that TCz-F-Cm is a RTP material with ultralong afterglow. While TCz-F-Lab shows blue fluorescence and greenish-yellow phosphorescence before and after turning off excitation source, the afterglow is much shorter than that of TCz-F-Cm, and the afterglow gradually disappears after 1.0 s. The powder of TCz-F-Cm exhibits prompt fluorescence at 440 nm, which is red-shifted by 20 nm than TCz-H-Cm. (Fig. 2c, d). However, TCz-F-Lab and TCz-H-Lab emit blue fluorescence at 400 nm and 390 nm, respectively. As shown in delayed PL spectra, the main peak of TCz-F-Cm centers at 556 nm and the delayed emission of TCz-H-Cm locates at 575 nm. While, the delayed PL spectra of TCz-F-Lab and TCz-H-Lab are blue-shifted to 515 nm and 530 nm. Their phosphorescence quantum yields are calculated to be 7.4%, 4.6%, 0.8%, 0.7% for TCz-F-Cm, TCz-H-Cm, TCz-F-Lab, and TCz-H-Lab, respectively. For TCz-OH, it exhibits only prompt PL, revealing that it is a fluorescent material. Temperature-dependent PL spectra were performed to further confirm their phosphorescence behaviors. With an increase of temperature from 50 K to 400 K, the PL intensities of TCz-F-Cm and TCz-H-Cm decrease bit by bit because of a higher possibility of energy loss through molecular motion at elevated temperature (Supplementary Figs. 19, 20). In addition to the fluorescent emission at 380-480 nm, phosphorescence signals are observed between 500 nm and 640 nm at cryogenic temperature, which is in good agreement with their delayed PL spectra. The lifetime of TCz-F-Cm decreases from 727 ms to 268 ms with an increment of temperature (Supplementary Table 3). TCz-H-Cm exhibits a similar phenomenon and its lifetime reduces from 128 ms to 11 ms. In contrast, TCz-OH-Cm shows no obvious lifetime change and the value always remains at the nanosecond level (Supplementary Fig. 21). Similarly, the lifetime of TCz-F-Lab and TCz-H-Lab decreases from 48.65 ms to 21.72 ms and from 29.93 ms to 0.96 ms during elevation of temperature, respectively, which are much shorter than those emitters based on commercial Cz (Supplementary Figs. 22, 23). Above results suggest that although the isomer (1H-Benz[f]indole) of Cz-Cm can improve RTP emission efficiency and elongate lifetime dramatically, the different emission behaviors of Cz-functionalized emitters are dominated by their various push-pull electronic effect in our system. The steady-state PL spectra of TCz-F-Cm and TCz-H-Cm in THF solution at 77 K were also collected in vacuum (Supplementary Fig. 24). All the fine vibrational structures can be seen clearly at low temperature and the corresponding lifetime of TCz-F-Cm and TCz-H-Cm in solution was evaluated to be 160 ms and 822 ms, respectively. For practical applications, the light-emitting materials are commonly applied as solid thin films. As such, poly(vinyl alcohol) (PVA) film containing TCz-F-Cm was fabricated by dissolving TCz-F-Cm in PVA solution (weight ratio of TCz-F-Cm to PVA is 1%) and then coating the resultant solution on a slide’s surface. The as-prepared film shows almost 100% light transmission and emits blue emission with a lifetime of 9.80 ms (Supplementary Fig. 25). Such behavior verifies its phosphorescent characteristic in the PVA film.

### Optical waveguide

**Fig. 3:**
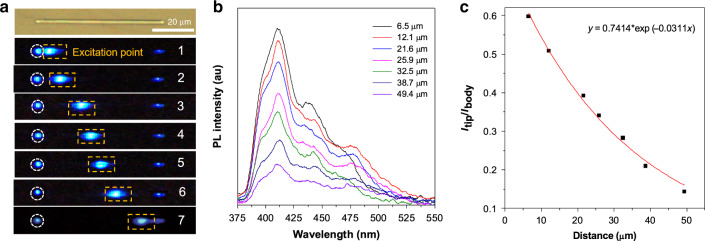
Optical waveguide performance of TCz-F-Cm. **a** The bright-field microscopic image of a microrod of TCz-F-Cm with a scale bar of 20 μm. Fluorescence microscopic image of this microcrystal with a focused laser (*λ* = 375 nm) at different excitation positions. **b** The corresponding PL spectra collected at the tips of (1)–(7) with a distance of 6.5–49.4 μm from the excitation point. **c** The related curve of the ratio of the PL intensity at the tip and the excitation point, respectively, versus distance *D* at 420 nm (*y* = 0.7414 × exp(−0.0311*x*)). The curve is fitted by an exponential decay function *I*_tip_/*I*_body_ = *A*exp(−α*D*).

Crystalline state is a key way to achieve triplet emission for most organic phosphorescent materials. Here, taking TCz-F-Cm as an example, its optical waveguide properties were conducted in crystals. The rod-like microcrystals were readily fabricated in a mixture of dichloromethane and ethanol. To investigate the light transmission behavior, microcrystal with a length of 56 μm was selected (Fig. 3a). The as-prepared microcrystal emits strong blue emission under 375 nm laser irradiation. When placing the laser source at distances (*D*) of 6.5, 12.2, 21.6, 25.9, 32.5, 38.7, and 49.4 μm from the left tip of the rod, strong blue emission at the brim is observed. The PL intensity at the left edge gradually decreases with an increase of distance from excitation point to edge (Fig. 3b). No obvious phosphorescence is observed in the microcrystals. As reported before, the triplet excitons can migrate with diffusion lengths of more than 10 μm in single crystals^42^. Thus, the triplet excitons are readily transferred to the microcrystal surface and then released to outside environment to result in deactivation. Moreover, the defects in crystals is another quenching process for triplet excitons^43^. Therefore, only fluorescence is observed in these microcrystals. The related curve of the ratio of the PL intensity at 415 nm at the tip and the excitation point versus distance D is depicted in Fig. 3c. The curves can be fitted by a single-exponential decay function *I*_tip_/*I*_body_ = *A*exp(−αD). The value of the optical-loss coefficient (*α*) is calculated to be as low as 0.031 dB μm^−1^, suggesting the good optical waveguide performance of these as-prepared single-crystalline organic microrods.

In addition, when the crystal size of TCz-F-Cm increases to millimeter level, they exhibit significant difference in optical property as compared to their components with micrometer sizes. As shown in Supplementary Fig. 26, a crystal with a length of 1.8 mm is selected for research. When changing the location of excitation point, sky-blue emission is observed at the right edge. Meanwhile, some new peaks at yellow region are recorded. As discussed above, they should be attributed to phosphorescent emission. When the crystal size is much bigger than the diffusion distance, the triplet excitons are difficult to reach the crystal surface. Thus, some triplet excitons are survived after removal of excitation source. The decay function of the ratio of the PL intensity at the tip and the excitation point versus distance is fitted to be *y* = 0.047 × exp(−0.00218*x*). The optical waveguide experiment reveals that when the crystals grow bigger, light scattering is easy to occur, which is not favorable for optical propagation.

### Electroluminescence

**Fig. 4:**
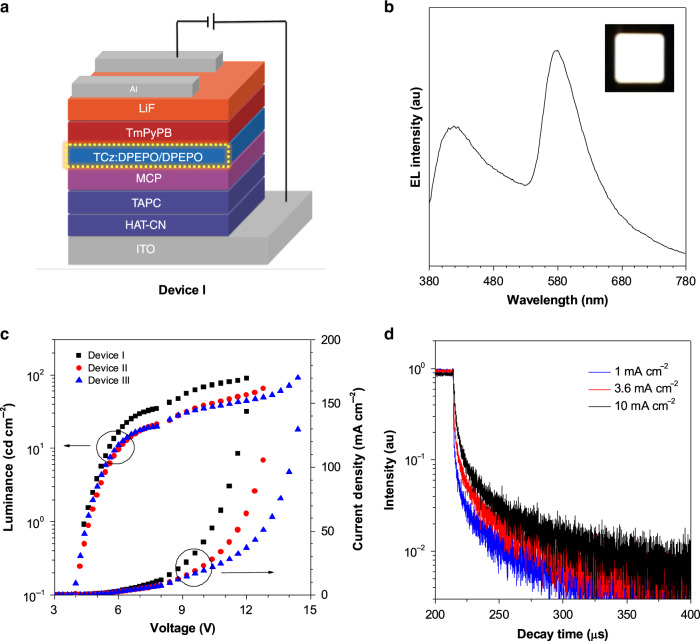
White light-emitting devices. **a** Configuration of device I. **b** The EL spectrum of device I at current density at 3.6 mA cm^−2^. Inset: photo of device I. **c** The luminance-voltage-current density curves of the devices I. **d** Transient EL decay curves at 580 nm of Device I at different current densities.

Inspired by the intriguing RTP and strong blue fluorescence of TCz-F-Cm/Lab, we made an attempt to fabricate phosphorescent electroluminescence (EL) devices (Fig. 4). Detailed fabrication processes and device configurations can be found in the Supporting Information. Here, TCz-F-Cm was selected as an emitter material due to its relatively high emission efficiency. To minimize the effect of bimolecular quenching such as triplet-triplet annihilation, TCz-F-Cm was doped in a host DPEPO (bis[2-((oxo)diphenylphosphino)phenyl] ether, 3.00 eV) with a high triplet-energy at low concentrations of 3, 6, and 10 wt% (device I, II and III, respectively). Meanwhile, 9,9′-(1,3-phenylene)bis-*9H*-carbazole (mCP, 2.90 eV) and DPEPO were utilized as exciton blocking layer to avoid exciplex production in the structures. The EL devices are turned on at 4.6−4.8 V. Surprisingly, a deep-blue emission peak at 420 nm and an orange peak at 580 nm are detected. With an increase of current density, the orange emission peak becomes predominant (Supplementary Fig. 27). Consequently, white-light emission was generated with CIE coordinates of (0.357, 0.317), (0.338, 0.307), and (0.347, 0.327) and maximum external quantum yield of 0.33% (Supplementary Table 4) at different current densities (3.6, 6.3, and 16.8 mA cm^−2^ for device I, II, and III, respectively) (Supplementary Figs. 28, 29).

These amazing results stimulate us to study the associated emission mechanism. The deep-blue emission peak of TCz-F-Cm at 420 nm should be ascribed to radiative transition from S_1_ → S_0_, as it is quite close to the PL peak and no other functional materials in the devices show visible emission. To further dig into the origin of the orange emission, transient EL decay curves at 580 nm of device I were measured at current density (*J*) of 1, 3.6 and 10 mA cm^−2^ (Fig. 4d). In contrast to the transient EL decay lifetime of singlet-exciton deactivation (at the order of nanosecond), the lifetime of device I at 580 nm is measured to be 1.17, 3.43, and 9.83 μs at 1, 3.6, and 10 mA cm^−2^, respectively (Supplementary Table 5), indicating a relatively long transition process from triplet state to S_0_. Crystalline TCz-F-Cm and the dimer of TAPC both emit phosphorescence at about 580 nm. Therefore, it is very difficult to infer which one contributes orange phosphorescence in the devices. As a control experiment, devices IV–VIII without TAPC were fabricated. Only blue fluorescence was observed in devices IV–VIII (Supplementary Tables 6, 7 and Fig. 30), suggesting that the orange emission is originated from T_1_ → S_0_ radiative transition of the dimer of TAPC. Among them, Device IV with the structure of indium tin oxide (ITO)/hexaazatriphenylenehexacabonitrile (HATCN, 5 nm)/*N*, *N*’-bis(naphthalen-1-yl)-*N*, *N*’-bis(phenyl) benzidine) (NPB, 40 nm) /mCP (5 nm)/3 wt% TCz-F: DPEPO (20 nm)/DPEPO (10 nm)/1,3,5-tris(*N*-phenylbenzimidazol-2-yl) benzene (TPBi, 30 nm)/LiF (1 nm)/Al exhibits a maximum external quantum efficiency (*η*_ext_) of 1.06% with a deep-blue emission at CIE coordinate of (0.155, 0.101). It’s a pity that we fail to obtain a phosphorescent OLED, but we have made an effort to fabricate white-light emitting EL devices through hybrid of blue fluorescence and yellow phosphorescence from a single molecule.

## Discussion

Molecular interactions and the packing motifs in crystals are strongly associated with their photophysical properties, thus we analyzed the crystal structures of TCz-F, TCz-H, and TCz-OH in Supplementary Figs. 31–33. All the molecules adopted a twisted conformation and many C-H···π interactions are observed in these crystals. The distances are calculated to be 2.828 Å, 2.811 Å and 2.863 Å for TCz-F, TCz-H and TCz-OH, respectively. In addition to C-H···π interactions, strong C-H···F interactions (*d* = 2.66 and 2.55 Å) exist in crystals of TCz-F. Intermolecular π···π stackings between two neighboring carbazole units are observed in crystals of TCz-H and TCz-OH, this interaction is not found in TCz-F. Such condensed molecule packing and strong intermolecular interactions are beneficial for efficient electron transfer between their molecular orbitals. Moreover, a flower pattern is established in the crystal structure of TCz-F. Other two molecules form a grid-like pattern. In brief, all these twisted conformations and multiple intermolecular interactions help to restrict molecular motions to activate the radiative transition. When the crystalline samples are ground to amorphous state with a mortar and pestle, their RTP disappears.

To get insight into the different RTP features of TCz-F, TCz-H, and TCz-OH, the deactivation processes from the excited state to the ground state were investigated. The fluorescence and phosphorescence rate constants of TCz-F, TCz-H and TCz-OH were collected and calculated (Supplementary Table 8). The rate constants of TCz-H from S_1_ to S_0_ (*k*_r_^Fluo^) and the intersystem crossing from S_1_ to T_1_ (*k*_ISC_) are 2.99 × 10^6^ and 8.08 × 10^6^ s^−1^, respectively. The *k*_r_^Fluo^ and *k*_ISC_ values of TCz-F increase to 4.03 × 10^7^ and 1.01 × 10^7^ s^−1^, demonstrating that introduction of fluoro group can enhance emission efficiency and possibility of intersystem crossing. The *k*_r_^Phos^ values from T_1_ to S_0_ of hydrocarbon molecules are generally in the range of 10^−1^–10^2^ s^−1^^44^. As a result, it is reasonably inferred that emitters with appropriate *k*_r_^Phos^ values are able to show persistent RTP if non-radiative transition is efficiently inhibited. The τ (727 ms), *k*_r_^Phos^ (0.12 s^−1^) and *k*_nr_^Phos^ (1.51 s^−1^) of TCz-F are comparable to those of recently reported RTP materials (τ (520 ms), *k*_r_^Phos^ (0.027 s^–1^), and *k*_nr_^Phos^ (1.90 s^–1^))^45^. For TCz-H, the corresponding *k*_r_^Phos^ and *k*_nr_^Phos^ are 1.93 s^–1^ and 40.08 s^–1^, respectively. The high *k*_nr_^Phos^ value of TCz-H is attributed to its short lifetime (0.023 s) at ambient temperature, which leads to its low-efficient RTP.

**Fig. 5:**
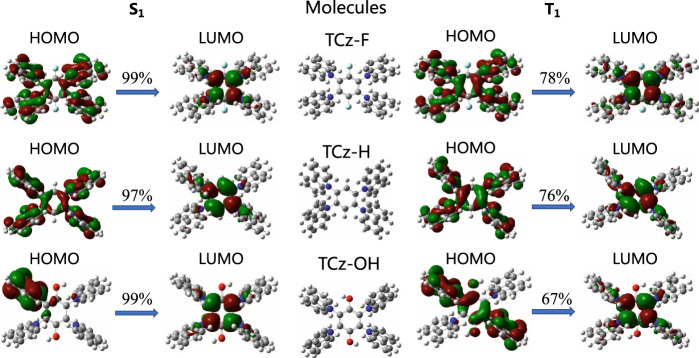
Molecular orbital. Calculated transition orbitals of the S_1_ and T_1_ states at the optimized S_1_ and T_1_ geometries for TCz-F, TCz-H, and TCz-OH, respectively, in the solid phase.

**Fig. 6:**
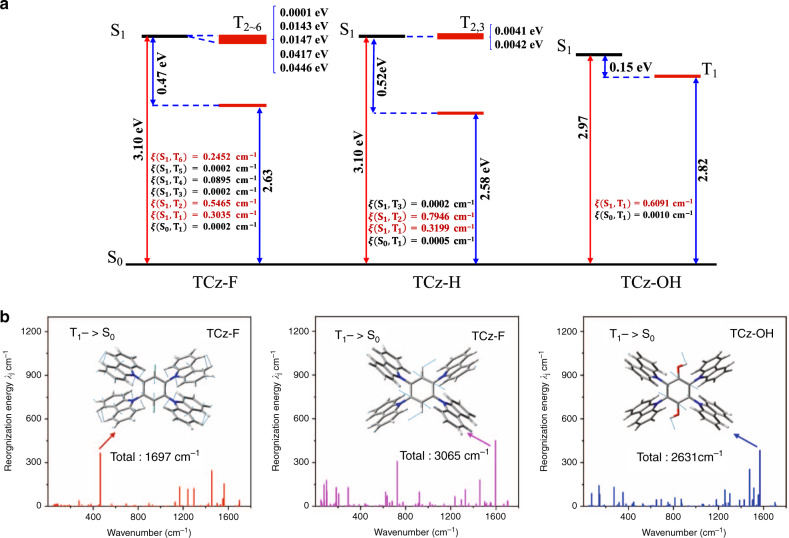
Calculated energy diagram. Calculated **a** energy diagram and spin-orbital coupling (*ξ*) at the S_1_-geometry; **b** The normal mode reorganization energy between T_1_ and S_0_ for TCz-F, TCz-H and TCz-OH in the solid state.

Moreover, theoretical investigations of the different emission behaviors of TCz-F, TCz-H and TCz-OH were carried out in both solution and solid state by using density functional theory (DFT) and time-dependent DFT (TD-DFT) (Supplementary Table 9). It is known that phosphorescence relies on two main processes including the effective intersystem crossing from S_1_ to the triplet states (T_*n*_) and the competition between the radiative and non-radiative processes from T_1_ to S_0_. Thus, the geometries of these three molecules at the S_0_, S_1_ and T_1_ state were optimized in both solution and solid phase, and their electronic structure properties were examined based on the optimized geometries, including the transition orbitals, vertical excitation energy, spin-orbit couplings between the low-lying singlet and triplet states, and the reorganization energy between T_1_ and S_0_ (Fig. 5 and Supplementary Figs. 34, 35). It is found that the highest occupied molecular orbital (HOMO) of TCz-F and TCz-H resides on the whole molecule in both S_1_ and T_1_ states because of the electron-donating carbazole ring (Fig. 5). While the HOMO of TCz-OH mainly localizes on one carbazole unit in the S_1_ state, and two carbazole units and the central phenyl ring in the T_1_ state owing to its electron-deficient character. The lowest unoccupied molecular orbital (LUMO) of all molecules, on the other hand, centers on the phenyl core. Such a result reveals that these molecules exhibit obvious charge-transfer (CT) character, which result in their *k*_r_^Fluo^ of ~10^6–7^ s^−1^ from experimental measurement is one or two order of magnitude smaller than the common strong fluorescence molecules of 10^8^ s^−1^. As shown in Fig. 6a, for TCz-F and TCz-H, many triplet states are extremely close to the S_1_ and their spin-orbit couplings are considerable for TCz-F in the solid phase, which both facilitate the intersystem crossing from S_1_ to T_*n*_. In contrast, there is only one triple state below S_1_ and the energy gap of TCz-OH is relatively larger in the solid state, which results in less efficient intersystem crossing from singlet to triplet than the others. On the other hand, the spin-orbit coupling and the reorganization energy between T_1_ and S_0_ of TCz-F are both the smallest among the three systems in the solid state, which leads to the slowest the non-radiative decay process (Fig. 6b)^46,47^. While TCz-OH has the strongest spin-orbit coupling and large reorganization energy in the solid state, which speeds up the non-radiative decay process from T_1_ to S_0_. In addition, there are less intersystem crossing paths from S_1_ to T_*n*_ and fast non-radative decay process with much larger *ξ* (S_0_, T_1_), indicating that they are not likely to show phosphorescence in solution at room temperature. Comparison between the calculated data of TCz-F, TCz-H, and TCz-OH gives a conclusion that it is more likely for TCz-F to show phosphorescence in the solid state as its smallest energy gap between S_1_ and T_*n*_, weakest spin-orbit coupling between T_1_ and S_0_, and lowest reorganization energy. These factors together contribute to a more competitive radiative process from T_1_ to S_0_.

In summary, a series of commercial/lab synthesized carbazole-based emitters (namely TCz-F, TCz-H, and TCz-OH) were designed and synthesized to realize control of molecular emission from phosphorescence to fluorescence by gradual tuning substituent from fluoro to hydrogen and then to hydroxyl group. Meanwhile, their emission lifetime decreases from second to nanosecond level during this process. Crystals of TCz-F exhibit strong blue fluorescence with good optical waveguide performance. Upon removal of excitation light source, obvious phosphorescence is observed with a lifetime of 727 ms and 48.65 ms for TCz-F-Cm and TCz-F-Lab, respectively. Through HPLC analysis, such huge difference in their luminescence behavior is attributed to an isomer of commercial carbazole, which can dramatically improve their emission efficiency and lifetime. Theoretical research reveals that small energy gap between S_1_ and T_*n*_, weak spin-orbit coupling between T_1_ and S_0_ and low reorganization energy are favorable to enhance intersystem crossing and contribute to a more competitive radiative process from T_1_ to S_0_. Using TCz-F-Cm as emitting layer, blue and white light-emitting OLEDs were successfully fabricated.

## Methods

### Materials

All reagents and solvents were chemical pure (CP) grade or analytical reagent (AR) grade and were used as received unless otherwise indicated. Carbazole was prepared according to previous literature.

### Measurements

^1^H NMR and ^13^C NMR spectra were measured on a Bruker AV 400 spectrometer at 298 K. Absorption spectra were recorded on a Hewlett Packard 8453 UV–Vis spectrophotometer. High-resolution mass spectra (HRMS) were obtained on a GCT Premier CAB 048 mass spectrometer operated in MALDI-TOF mode. Fluorescent emission spectra were collected on a Horiba FluoroLog 3 fluorometer at 298 K. Solid state quantum yield was measured using a Hamamatsu C11347 Quantaurus-QY integrating sphere. High performance liquid chromatography (HPLC) was performed on an Waters 1525 HPLC. The running rate was 10 mL/min, and running buffer was 70% methanol and 30% water. The lifetime, steady state and low temperature photoluminescence spectra were measured on a Edinburgh FLS980 fluorescence spectrophotometer equipped with a continuous xenon lamp (Xe1), a microsecond pulsed xenon flash lamp (uF920) and a nanosecond flash lamp (nF920), respectively. Single crystal data were collected on a Bruker Smart APEXII CCD diffractometer using graphite monochromated Mo Kα radiation (*λ* = 0.71073 Å) or Cu Kα radiation (*λ* = 1.54184 Å). The thermogravimetric analysis (TGA) measurements were performed on a TA Instrument TGA Q5000.

### Synthesis of TCz-F and Tcz-OH

To a 100 mL flask, commercial/lab synthesized carbazole (2 g, 12 mmol) and 60% sodium hydride dispersion in mineral oil (24 mmol) were dissolved in dry DMF (20 mL) under nitrogen atmosphere in ice bath and the mixture was stirred for 30 min. Then hexafluorobenzene (0.372 g, 2.0 mmol) was slowly added and stirred at 70 °C until reaction completion (monitored by TLC). The reaction was quenched by water and filtered to give a white solid, then recrystallized with toluene and obtained needle-like crystal TCz-F (0.77 g, 50%). ^1^H NMR (400 MHz, CDCl_3_) *δ* = 7.76 (d, *J* = 6.4 Hz, 8H), 7.24 (d, *J* = 4.8 Hz, 8H), 7.14–7.11 (m, 16H) ppm. ^13^C NMR (100 MHz, CDCl_3_) *δ* = 138.97, 125.66, 124.77, 124.68, 124.02, 120.88, 120.08, 109.91 ppm. MALDI-TOF HRMS m/z calculated for C_54_H_33_F_2_N_4_ 775.2673 [M + H]^+^; found 775.2697 [M + H]^+^.

Then the filtrate was extracted with chloroform and dried over anhydrous Na_2_SO_4_. After removal of solvent under reduced pressure, the residue was recrystallized using toluene to give a white crystal of TCz-OH (0.3 g, 19%). ^1^H NMR (400 MHz, CDCl_3_) *δ* = 8.01 (s, 2H), 7.78 (d, *J* = 6.4 Hz, 8H), 7.25 (d, *J* = 6.0 Hz, 8H), 7.09–7.15 (m, 16H). ^13^C NMR (100 MHz, CDCl_3_) *δ* = 161.90, 138.32, 125.04, 123.37, 120.25, 119.91, 119.46, 109.28 ppm. MALDI-TOF HRMS *m/z* calculated for C_54_H_35_N_4_O_2_ 771.2827 [M + H]^+^; found 771.2813 [M + H]^+^.

### Synthesis of TCz-H

TCz-H was afforded as a white solid by following a route similar to that for TCz-F. ^1^H NMR (400 MHz, *d*^8^-THF) *δ* = 8.44 (s, 2H), 7.83 (d, *J* = 7.6 Hz, 8H), 7.55 (d, *J* = 8.4 Hz, 8H), 7.09 (t, *J* = 12.0 Hz, 8H), 7.02 (t, *J* = 8.0 Hz, 8H). ^13^C NMR (100 MHz, *d*^8^-THF) *δ* = 139.17, 133.96, 131.29, 124.73, 123.14, 119.45, 119.10, 109.14 ppm. MALDI-TOF HRMS m/z calculated for C_54_H_35_N_4_ 739.2862 [M + H]^+^; found 771.2840 [M + H]^+^.

## Supplementary information

Supplementary Information

## Data Availability

The data that support the findings of this study are available in Supplemenary Information and from the corresponding authors on reasonable request. The crystal structure data of TCz-F, TCz-H, and TCz-OH have been deposited in Cambridge Structural Database as CCDC 1999133, 1999134, and 1999135, respectively. These data can be obtained free of charge from The Cambridge Crystallographic Data Centre via www.ccdc.cam.ac.uk/data_request/cif.
