# Supplementary material for: Tuning molecular emission of organic emitters from fluorescence to phosphorescence through push-pull electronic effects
Source: Nat Commun. 2020 May 26;11:2617. doi: 10.1038/s41467-020-16412-4 (PMC7251133; doi:10.1038/s41467-020-16412-4)
Supplement: Supplementary file 1 — Supplementary Information [file 41467_2020_16412_MOESM1_ESM.pdf]

Tuning Molecular Emission of Organic Emitters from Fluorescence to Phosphorescence through Push-Pull Electronic Effects

Hai-Tao Feng,[1, 2, #] Jiajie Zeng,[3, #] Ping-An Yin,[3] Xue-Dong Wang,[5] Qian Peng,[4, *] Zujin Zhao,[3, *] Jacky W. Y. Lam[2] and Ben Zhong Tang[2, 3, *]

[1]Baoji AIE Research Center, Shaanxi Key Laboratory of Phytochemistry, College of Chemistry and Chemical Engineering, Baoji University of Arts and Sciences, Baoji 721013, China

[2]Department of Chemistry, Hong Kong Branch of Chinese National Engineering Research Center for Tissue Restoration and Reconstruction, Institute for Advanced Study, The Hong Kong University of Science & Technology, Clear Water Bay, Kowloon, Hong Kong, China

[3]Center for Aggregation-Induced Emission, SCUT-HKUST Joint Research Laboratory, State Key Laboratory of Luminescent Materials and Devices, South China University of Technology, Guangzhou, China

[4]Key Laboratory of Organic Solids, Beijing National Laboratory for Molecular Science, Institute of Chemistry, Chinese Academy of Sciences, Beijing 100190, China

[5]Institute of Functional Nano & Soft Materials (FUNSOM), Jiangsu Key Laboratory for Carbon-Based Functional Materials & Devices, Soochow University, Jiangsu 215123, P. R. China

Corresponding authors: Qian Peng (qpeng@iccas.ac.cn), Zujin Zhao (mszjzhao@scut.edu.cn), Ben Zhong Tang (tangbenz@ust.hk)

Supplementary Methods

Calculation: Theoretical investigations, including the geometrical optimization in the S_0, S_1 and T_1 states and the corresponding electronic transition properties on TCz-F and TCz-OH were performed in both solution and solid state by using density functional theory (DFT) and time-dependent DFT (TD-DFT) with PBE0/6-31G(d). The solvation effect and aggregation effect were considered by using the polarizable model (PCM)[1] and ONIOM method[2] with high-level QM and low-level MM layers in Gaussian 16 program [1], respectively. The UFF force field was used with the restrained electrostatic potential (RESP) partial charges for the MM treatment. The QM/MM computational models were built by digging a cluster from the X-ray crystal structures, and shown in Supplementary Figure 36 taking TCz-F an example. The spin-orbit couplings (SOC) between singlets and triplets were evaluated at (TD)PBE0/6-31G(d) level by using Beijing Density Function (BDF) program.[2-5] The reorganization energies were evaluated based on the normal modes' displacements and frequency in MOMAP program. [6, 7]

Device Configurations:

IV ITO/HATCN (5 nm)/NPB (40 nm)/mCP (5 nm)/3 wt% TCz-F: DPEPO (20 nm)/DPEPO (10 nm)/TPBi (30 nm)/LiF (1 nm)/Al

V ITO/HATCN (5 nm)/NPB (40 nm)/TcTa (5 nm)/3 wt% TCz-F: PPF (20 nm)/PPF (10 nm)/TPBi (40 nm)/LiF (1 nm)/Al

VI ITO/HATCN (5 nm)/NPB (40 nm)/TcTa (5 nm)/6 wt% TCz-F: PPF (20 nm)/PPF (10 nm)/TPBi (40 nm)/LiF (1 nm)/Al

VII ITO/HATCN (5 nm)/NPB (40 nm)/TcTa (5 nm)/9 wt% TCz-F: PPF (20 nm)/PPF (10 nm)/TPBi (40 nm)/LiF (1 nm)/Al

VIII ITO/HATCN (5 nm)/NPB (40 nm)/TcTa (5 nm)/12 wt% TCz-F: PPF (20 nm)/PPF (10 nm)/TPBi (40 nm)/LiF (1 nm)/Al

Supplementary References

1. Frisch, M. et al. Gaussian 16, revision A. 03. Gaussian Inc. (Wallingford CT, 2016).

2. Li, Z., Suo, B., Zhang, Y., Xiao, Y. & Liu, W. Combining spin-adapted open-shell TD-DFT with spin–orbit coupling. *Mol. Phys.* **111**, 3741 (2013).

3. Liu, W., Hong, G., Dai, D., Li, L. & Dolg, M. The Beijing four-component density functional program package (BDF) and its application to EuO, EuS, YbO and YbS. *Theor. Chem. Acc.* **96**, 75 (1997).

4. Liu, W., Wang, F. & Li, L. The Beijing Density Functional (BDF) Program Package: Methodologies and Applications. *J. Theor. Comput. Chem.* **02**, 257 (2003).

5. Hirao, K. & Ishikawa, Y. Recent Advances in Relativistic Molecular Theory. In Recent Advances in Computational Chemistry, World Scientific: Singapore. **5**, 257 (2004).

6. Reimers, J. R. *J. Chem. Phys.* **115**, 9103 (2001).

7. Niu, Y. et al. MOlecular MAterials Property Prediction Package (MOMAP) 1.0: a software package for predicting the luminescent properties and mobility of organic functional materials. *Mol. Phys.* **116**, 1078 (2018).

Supplementary Figures

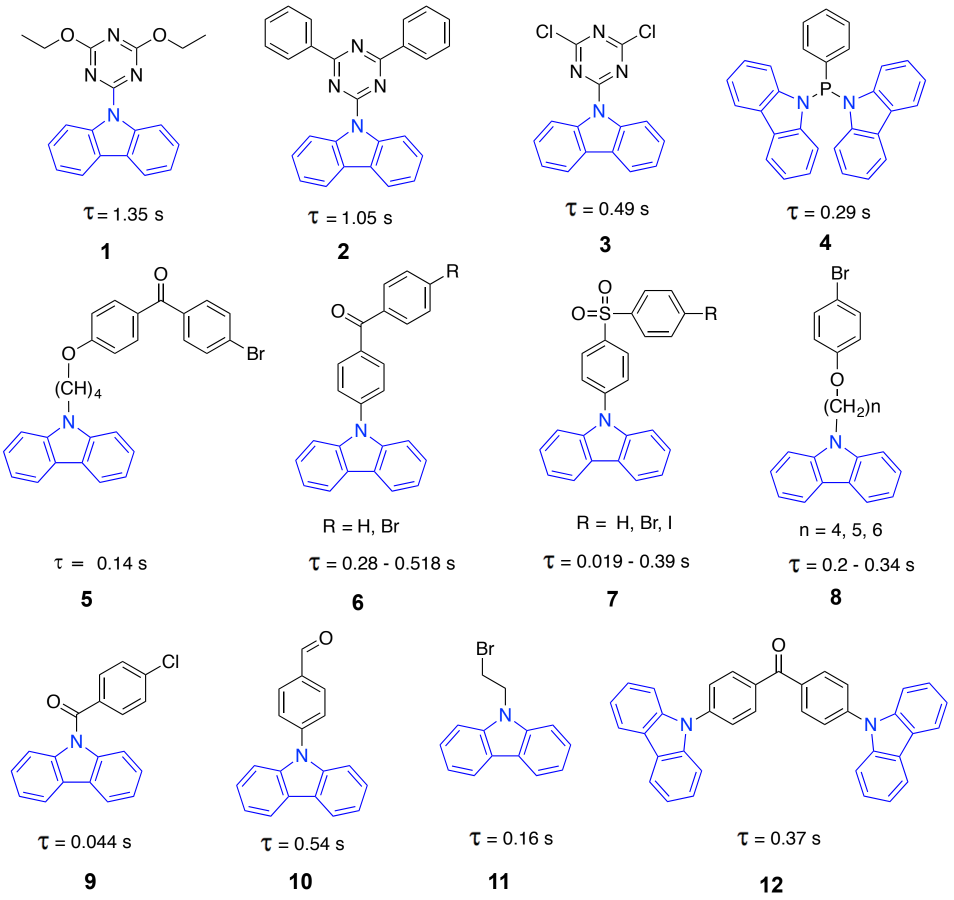

Supplementary Figure 1. Molecular structures of organic single-component commercial carbazole based RTP emitters.

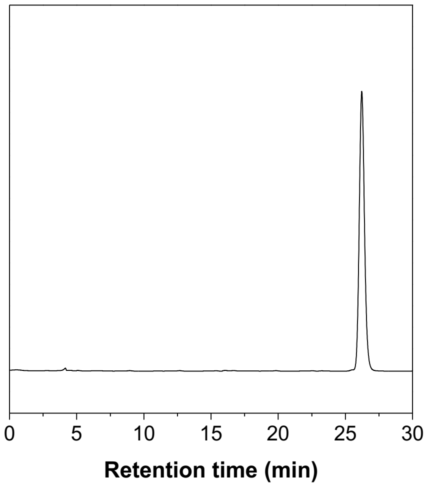

Supplementary Figure 2. High performance liquid chromatography diagrams of lab-synthesized carbazole.

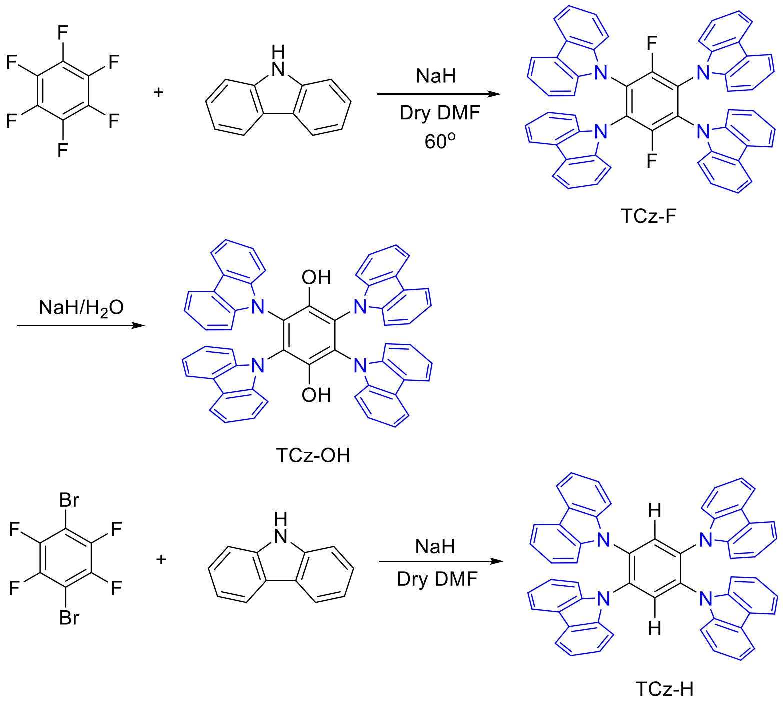

Supplementary Figure 3. The synthetic route of TCz-F, TCz-H and TCz-OH.

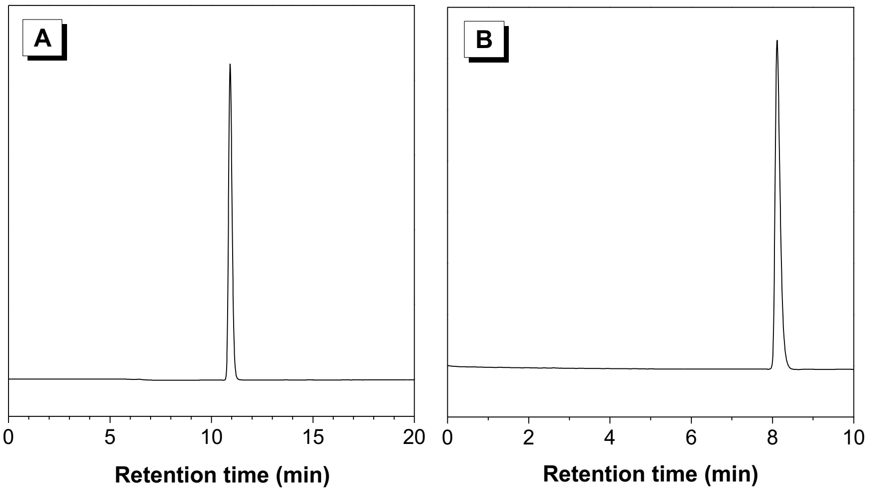

Supplementary Figure 4. High performance liquid chromatography diagrams of (A) TCz-F-Lab and (B) TCz-H-Lab.

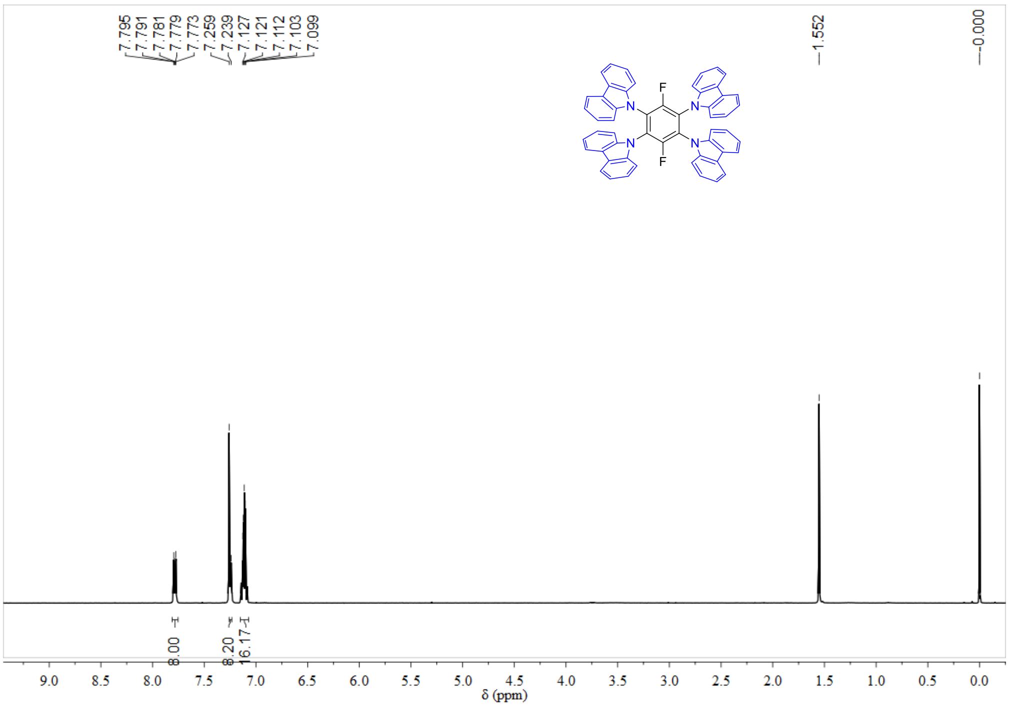

Supplementary Figure 5. ^{1}H NMR spectrum of TCz-F in $CDCl_3$.

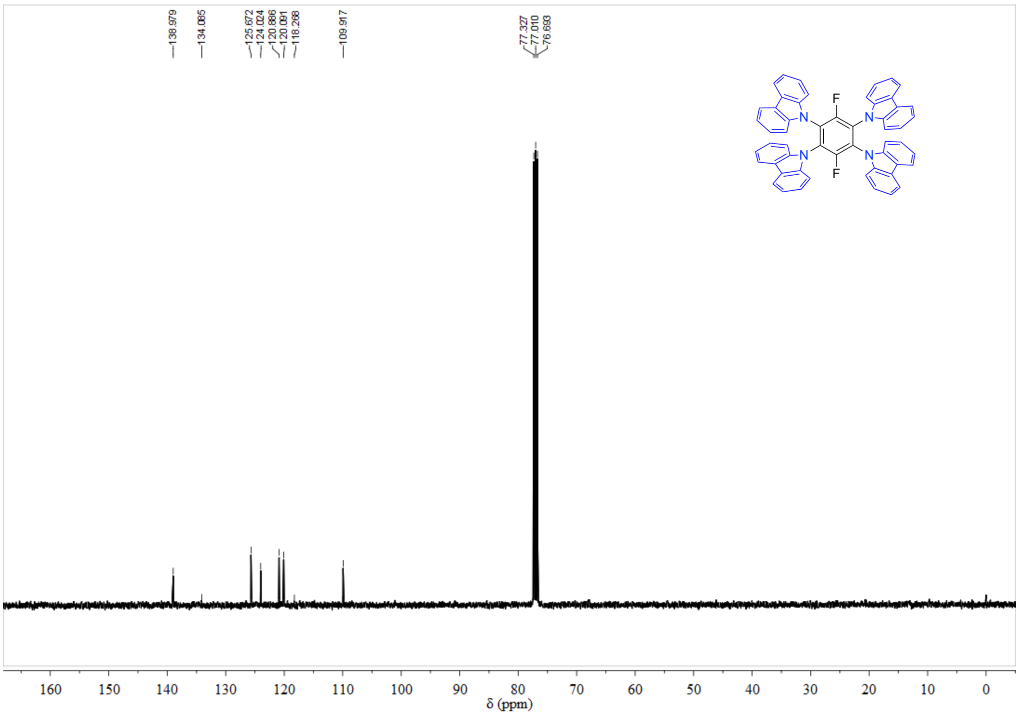

Supplementary Figure 6. ^{13}C NMR spectrum of TCz-F in $CDCl_3$.

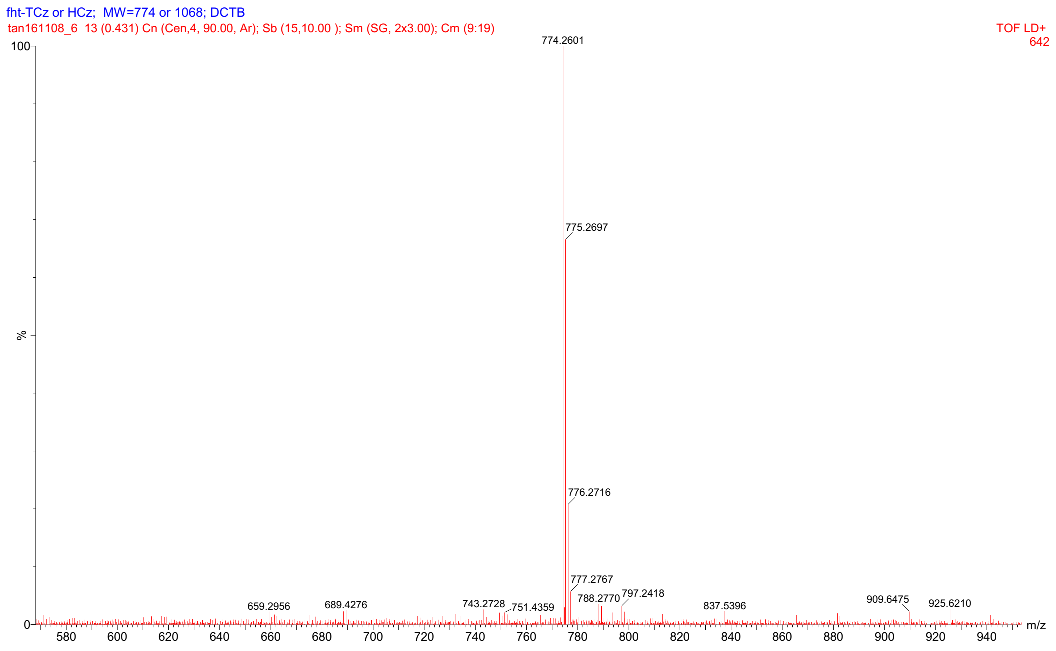

Supplementary Figure 7. HRMS spectrum of TCz-F.

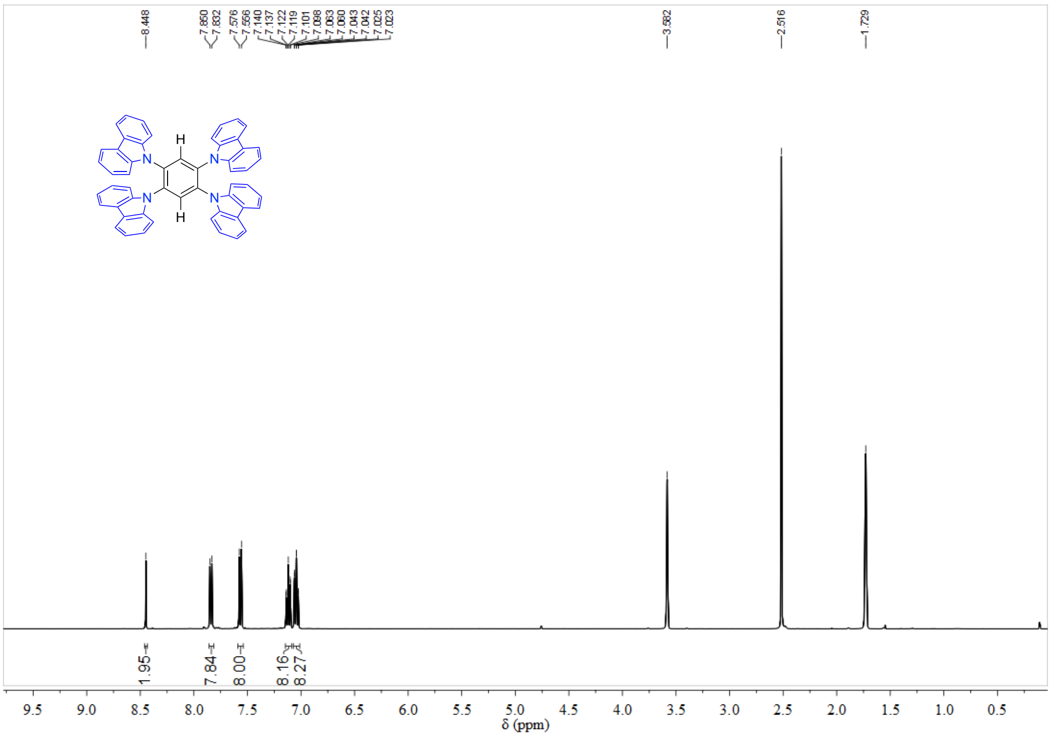

Supplementary Figure 8. ^{1}H NMR spectrum of TCz-H in d^6-THF.

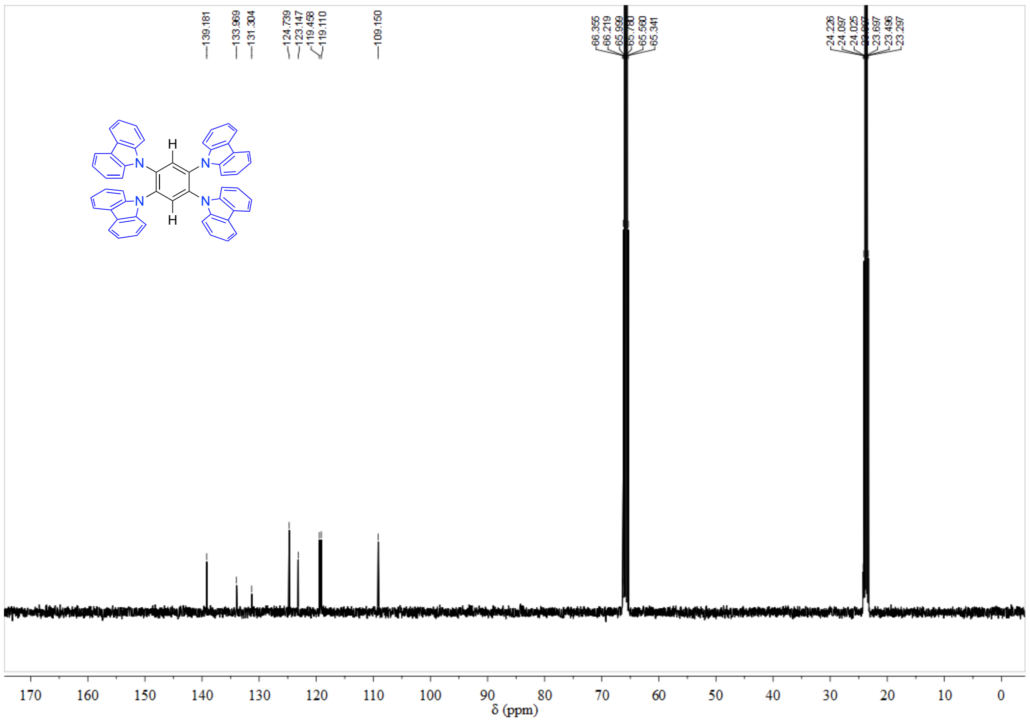

Supplementary Figure 9. ^{13}C NMR spectrum of TCz-H in d^6-THF.

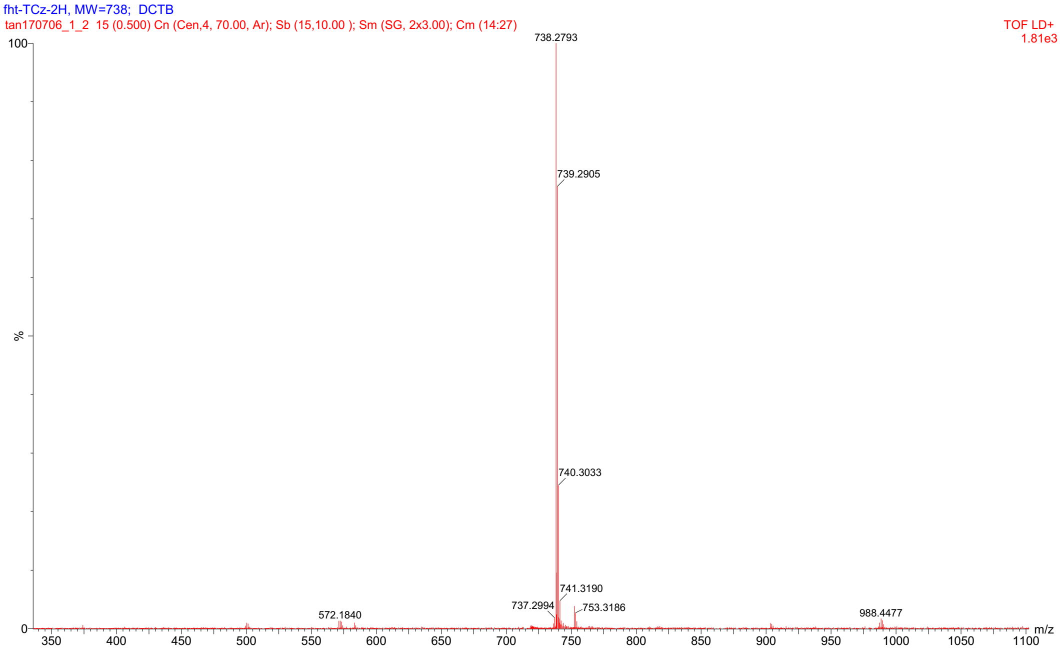

Supplementary Figure 10. HRMS spectrum of TCz-H.

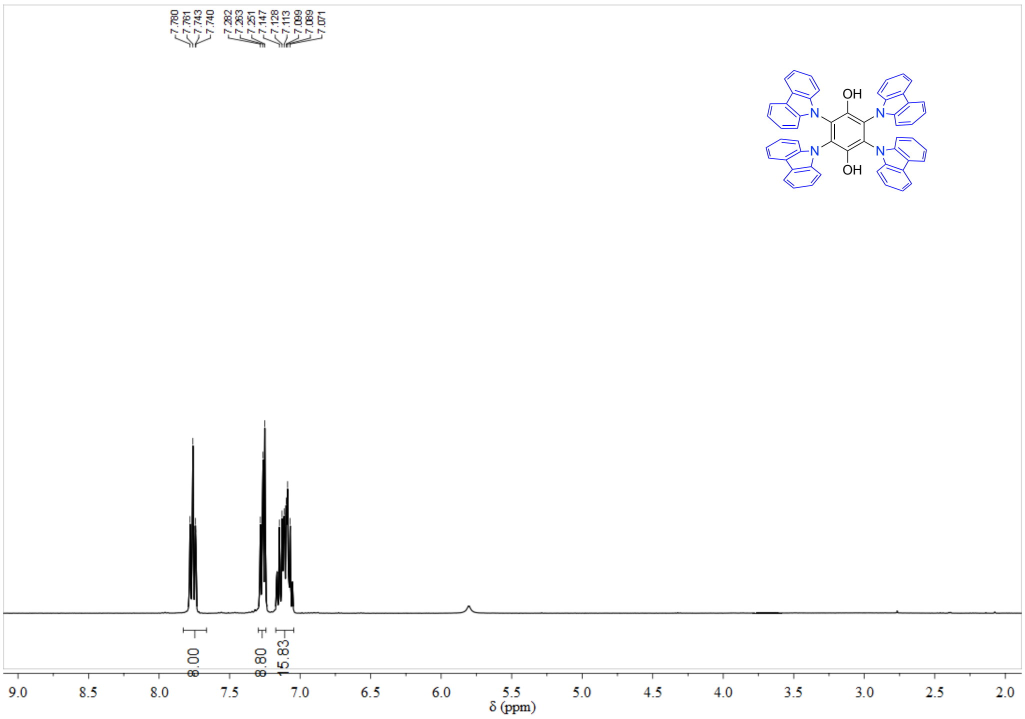

Supplementary Figure 11. ^{1}H NMR spectrum of TCz-OH in $CDCl_3$.

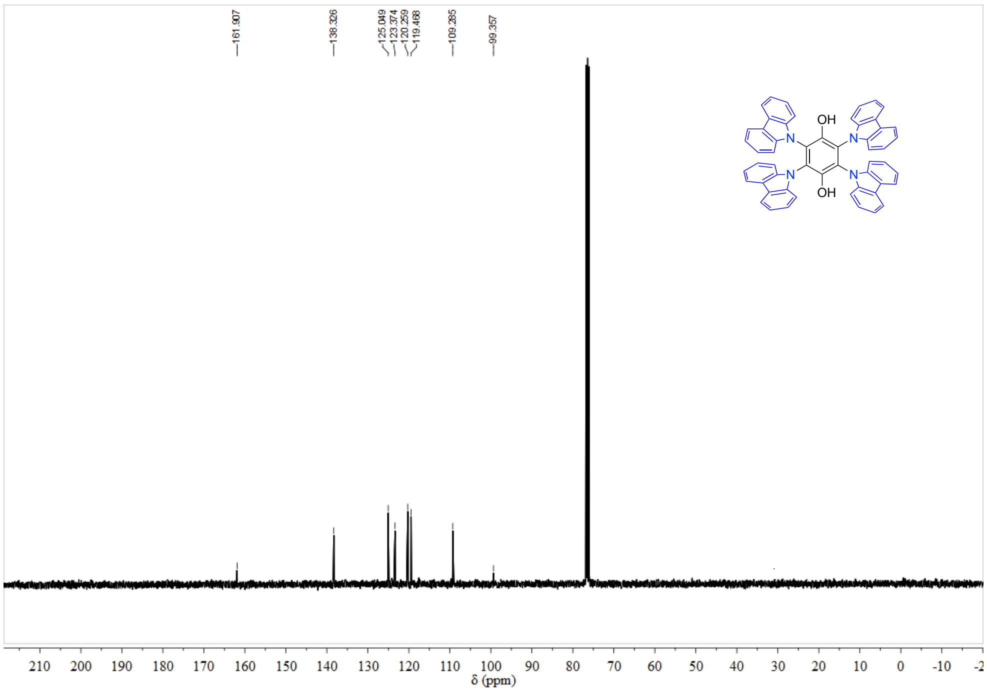

Supplementary Figure 12. ^{1}H NMR spectrum of TCz-OH in $CDCl_3$.

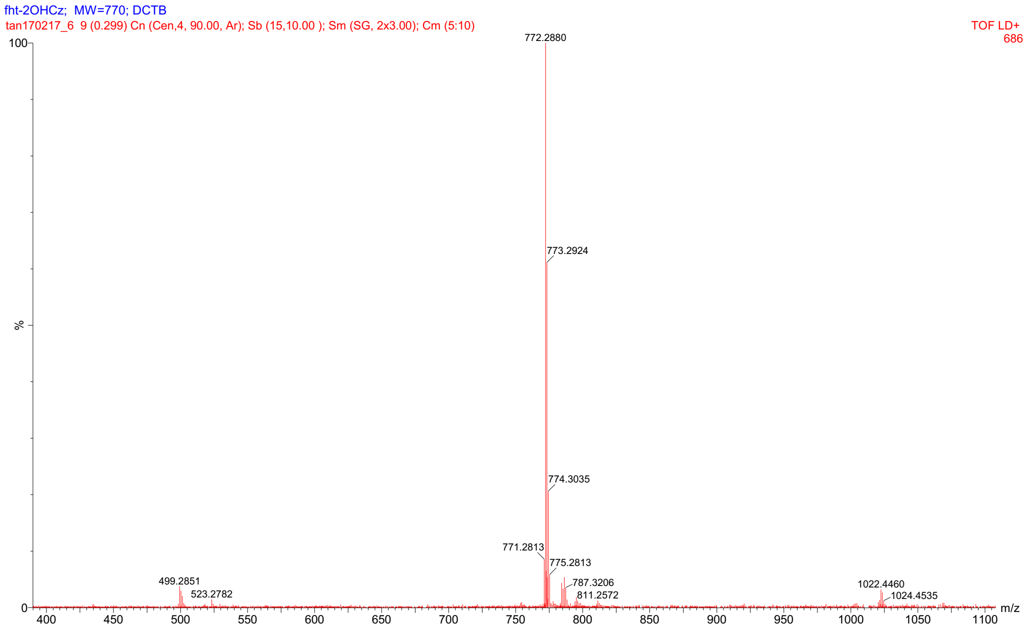

Supplementary Figure 13. HRMS spectrum of TCz-OH.

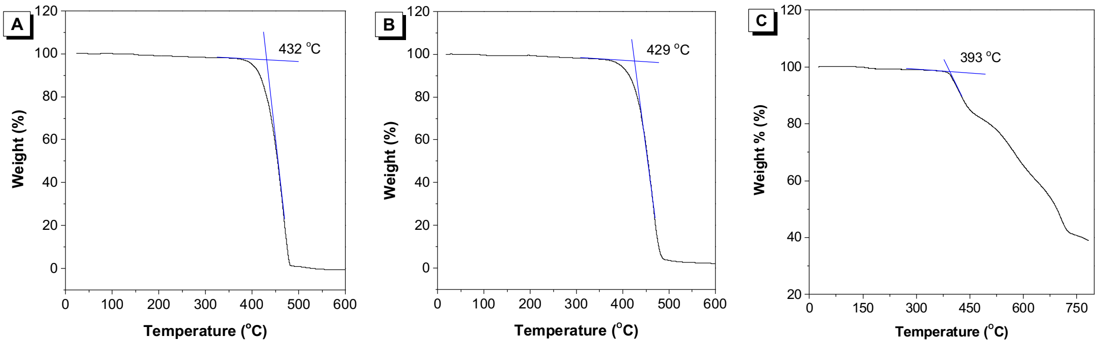

Supplementary Figure 14. TGA thermograms of (A) TCz-F-Cm, (B) TCz-H-Cm, (C) TCz-OH-Cm

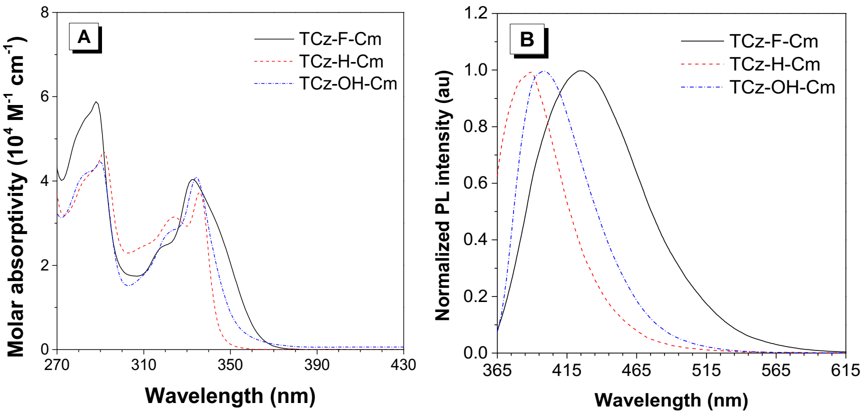

Supplementary Figure 15. (A) UV-vis absorption spectra and (B) normalized PL spectra of TCz-F-Cm, TCz-H-Cm and TCz-OH-Cm in THF solution. Concentration: 10 μM.

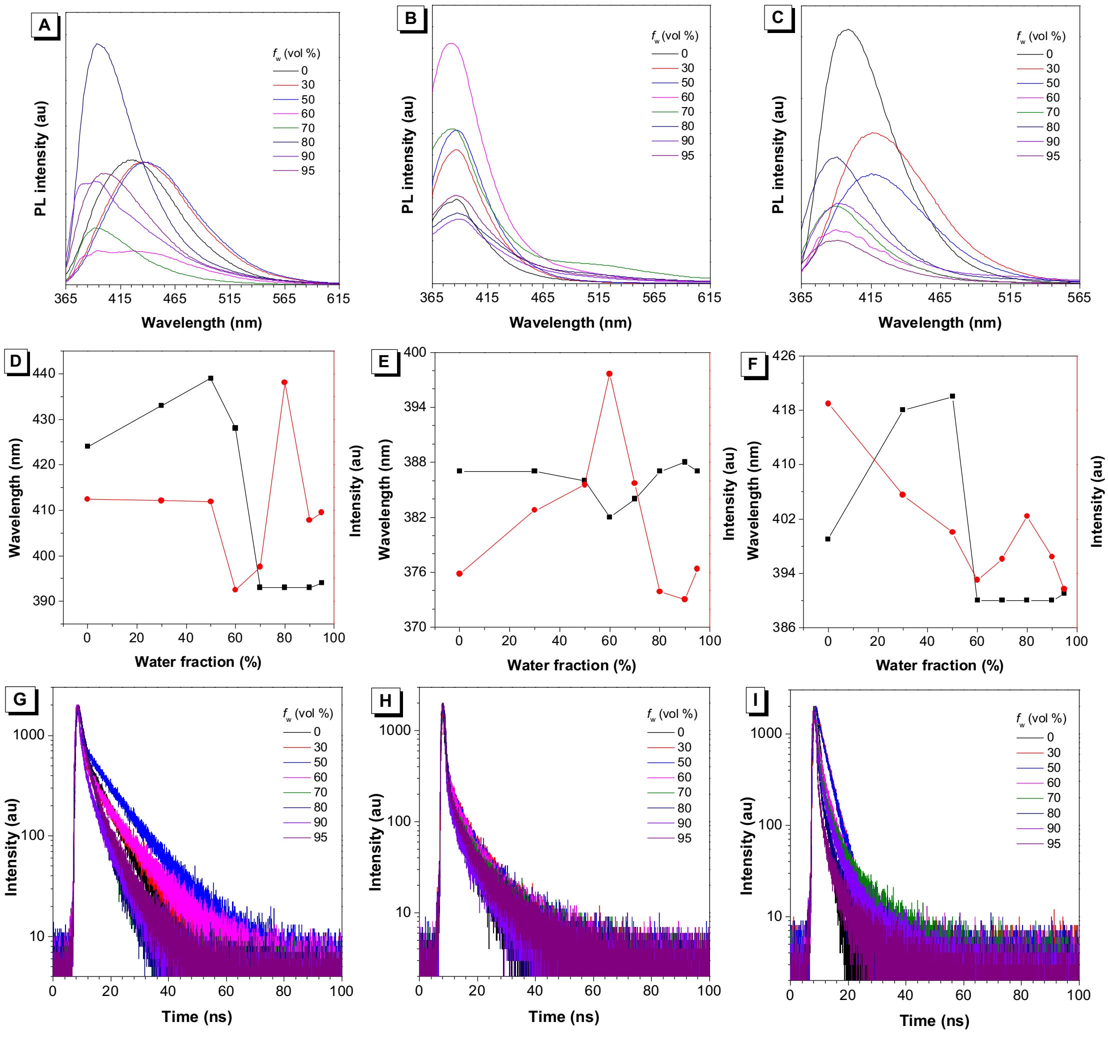

Supplementary Figure 16. Change of PL maximum, intensity and lifetime of (A, D, G) TCz-F-Cm, (B, E, H) TCz-H-Cm and (C, F, I) TCz-OH-Cm in THF and THF/water mixtures with different water fractions. Excitation wavelength: 350 nm. Concentration: 1.0 $\times 10^{-5}$ M.

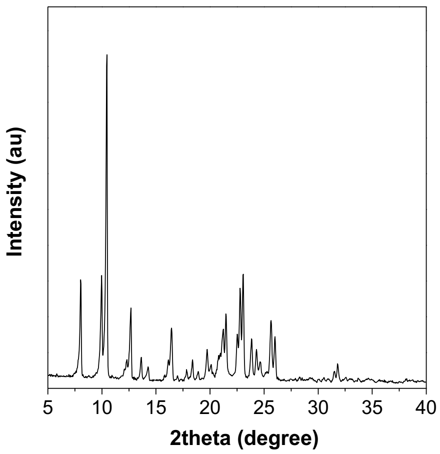

Supplementary Figure 17. Powder XRD pattern of TCz-F-Cm.

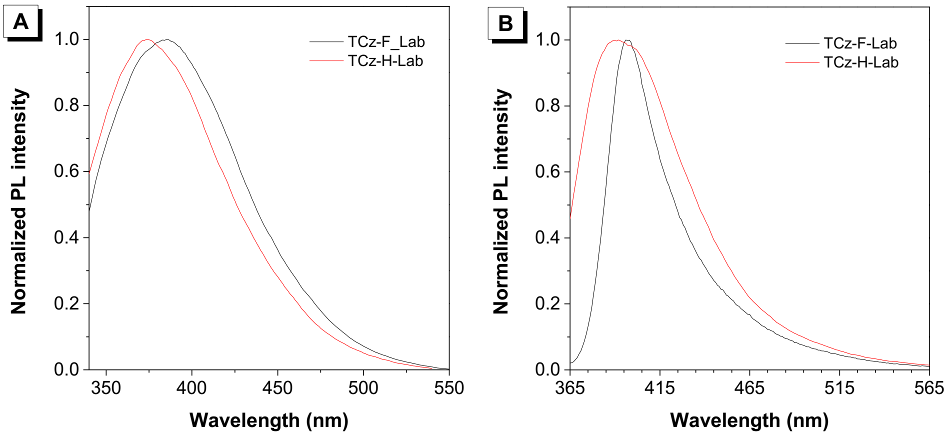

Supplementary Figure 18. Normalized PL spectra of TCz-F-Lab and TCz-H-Lab in THF solution (A) and solid state (B).

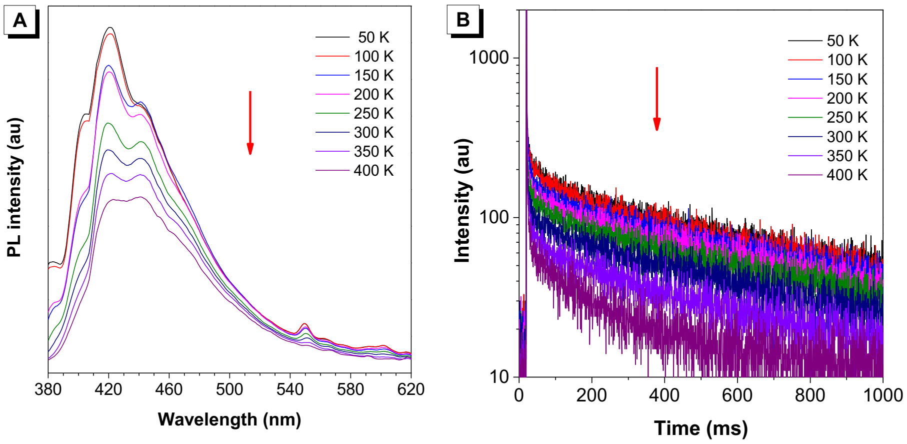

Supplementary Figure 19. (A) PL spectra of TCz-F-Cm at different temperature in the solid state. (B) Temperature-dependent PL decay spectra of TCz-F-Cm under nitrogen.

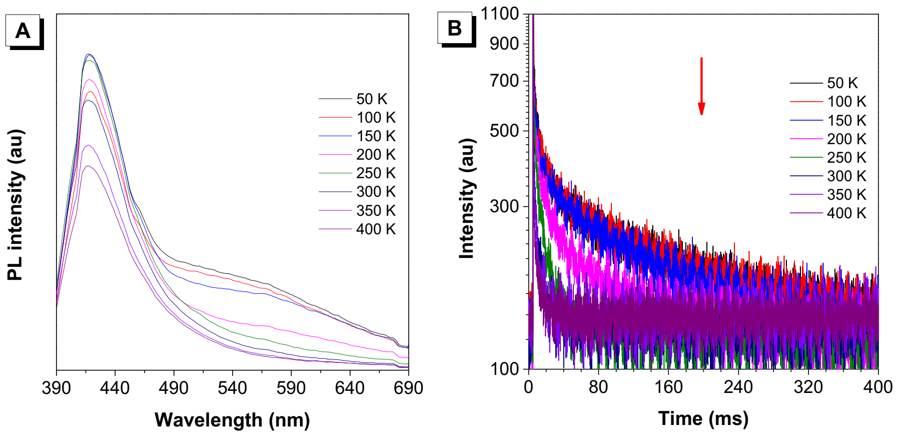

Supplementary Figure 20. (A) PL spectra of TCz-H-Cm at different temperature in the solid state. (B) Temperature-dependent PL decay spectra of TCz-H-Cm under nitrogen.

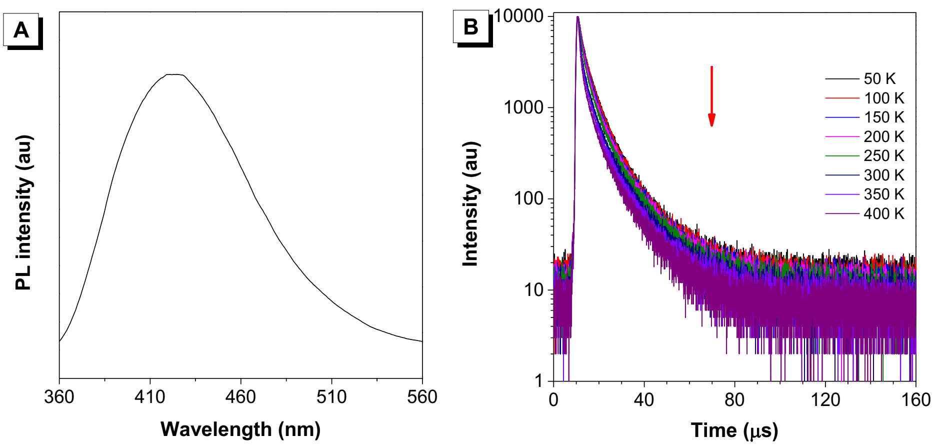

Supplementary Figure 21. (A) PL spectra of TCz-OH-Cm in the solid state. (B) Temperature-dependent PL decay spectra of TCz-OH-Cm under nitrogen.

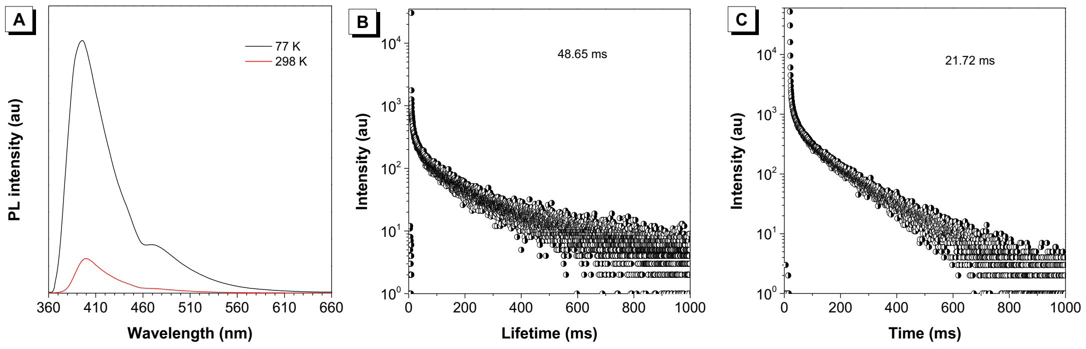

Supplementary Figure 22. (A) PL spectra of TCz-F-Lab at 77 K and room temperature in the solid state. Time-resolved PL decay curves of TCz-F-Lab at 77 K (B) and room temperature (C).

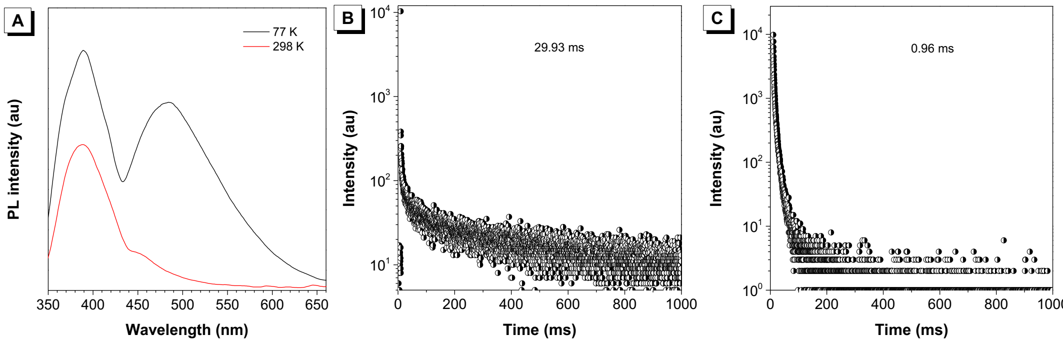

Supplementary Figure 23. (A) PL spectra of TCz-H-Lab at 77 K and room temperature in the solid state. (B) Time-resolved PL decay curves of TCz-H-Lab at 77 K (B) and room temperature (C).

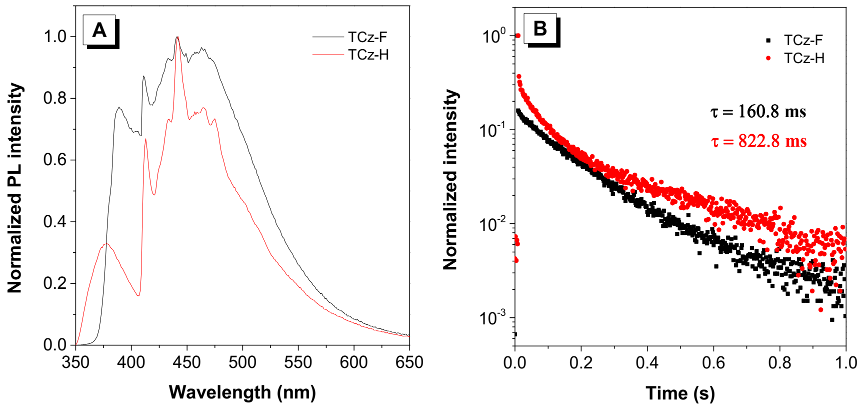

Supplementary Figure 24. (A) Prompt PL spectra of dilute THF solutions of TCz-F-Cm and TCz-H-Cm at 77 K. (B) Time-resolved PL decay curves at 466 nm. Concentration: 10 μM; Excitation wavelegnth: 330 nm.

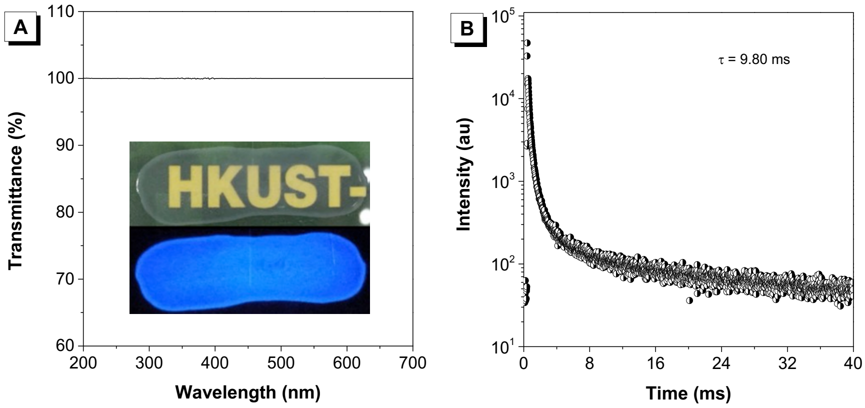

Supplementary Figure 25. (A) Transmittance spectrum of TCz-F-Cm doped PVA film. Inset: Photograph of TCz-F-Cm in PVA film deposited on a slide under daylight and 365 nm UV

irradiation. (B) PL decay spectra of TCz-F-Cm in PVA film in air at 580 nm. Excitation wavelegnth: 330 nm.

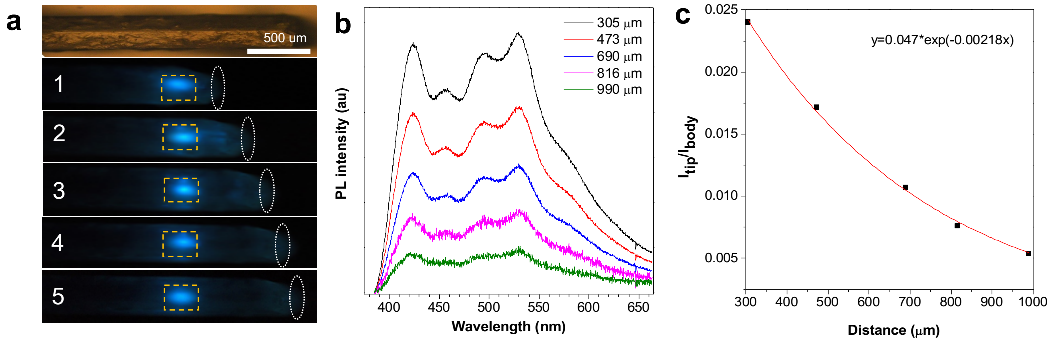

Supplementary Figure 26. (**a**) The bright-field microscopic image of a typical rod-like TCz-F-Cm crystal with a scale bar of 500 μm. (1–5) Fluorescence microscopic image of this long rod-like crystal with a focused laser (λ = 375 nm) at different excitation positions. (**b**) The corresponding PL spectra collected at the tips of (1)–(5) with a distance of 305–990 μm from the excitation point. (**c**) The related curve of the ratio of the PL intensity at the tip and the excitation point, respectively, versus distance at 423 nm ($y = 0.047 \times \exp(-0.00218x)$). The curves are fitted by an exponential decay function $I_{tip}/I_{body} = A\exp(-\alpha D)$.

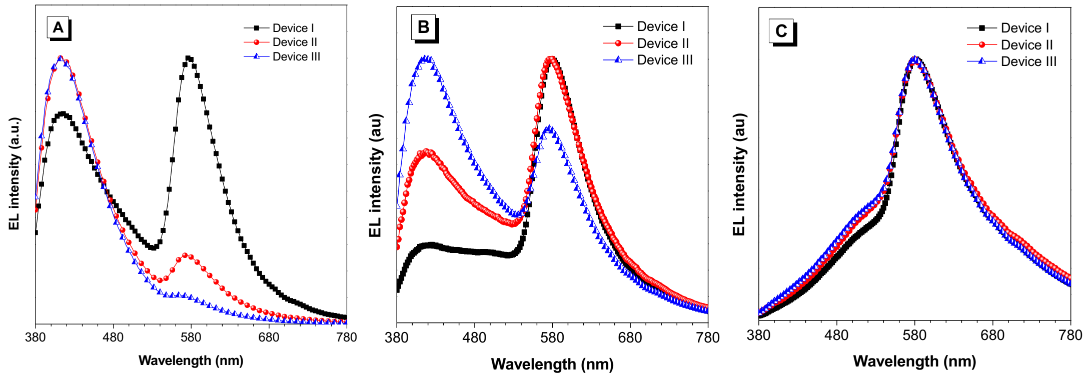

Supplementary Figure 27. The EL spectra of Device I, Device II and Device III at current density at (A) 1 mA cm^{-2}, (B) 10 mA cm^{-2} and (C) 100 mA cm^{-2}.

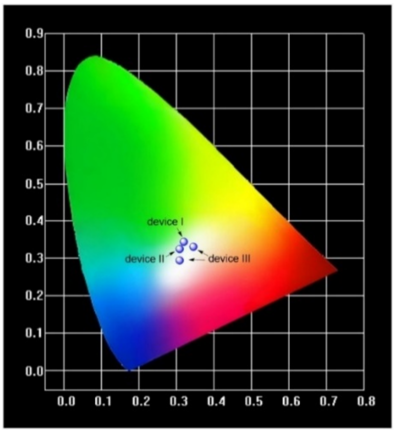

Supplementary Figure 28. CIE 1931 coordinates of Device I-III, I (0.35 0.32), II (0.33 0.31), III (0.34, 0.32).

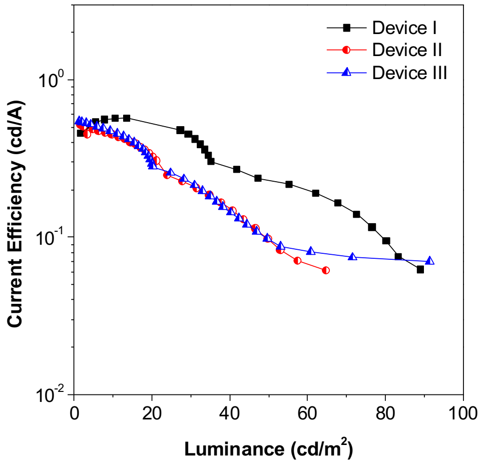

Supplementary Figure 29. The current efficiency-luminance curves of the devices.

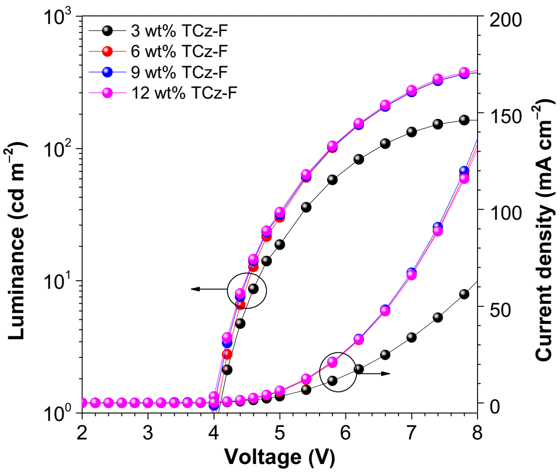

Supplementary Figure 30. The luminance-voltage-current density curves of devices IV-VIII.

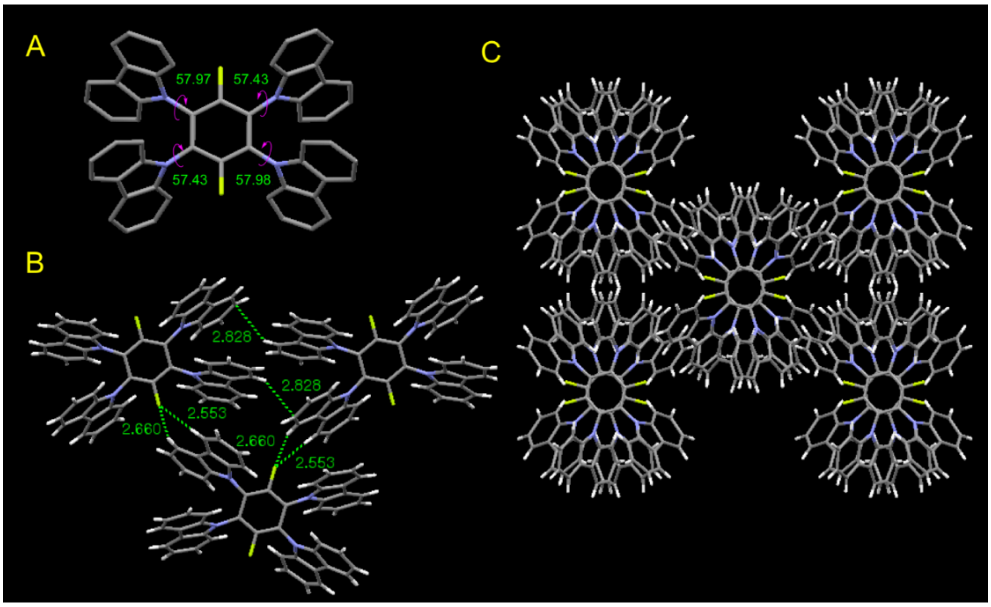

Supplementary Figure 31. Single crystal structure of TCz-F-Cm and associated intermolecular interactions. Hydrogen atoms are omitted for clarity.

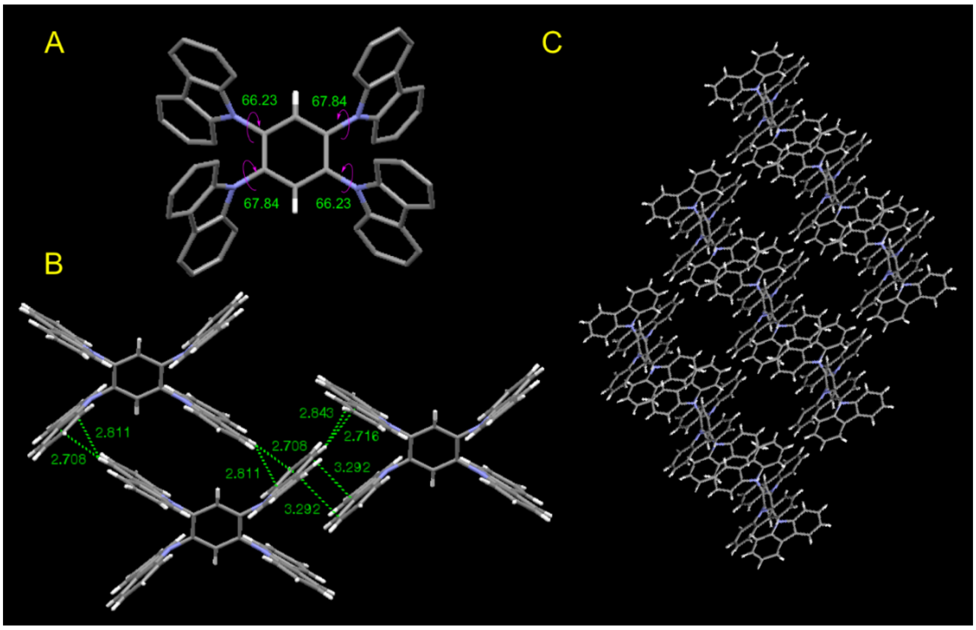

Supplementary Figure 32. Single crystal structure of TCz-H-Cm and the associated intermolecular interactions. Hydrogen atoms are omitted for clarity.

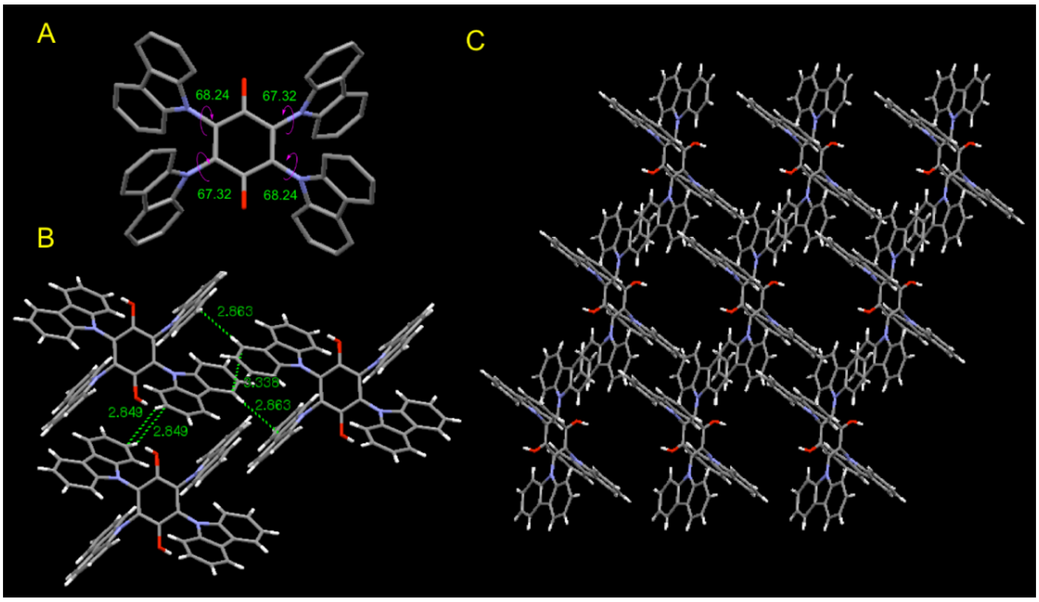

Supplementary Figure 33. Single crystal structure of TCz-H-Cm and associated intermolecular interactions. Hydrogen atoms are omitted for clarity.

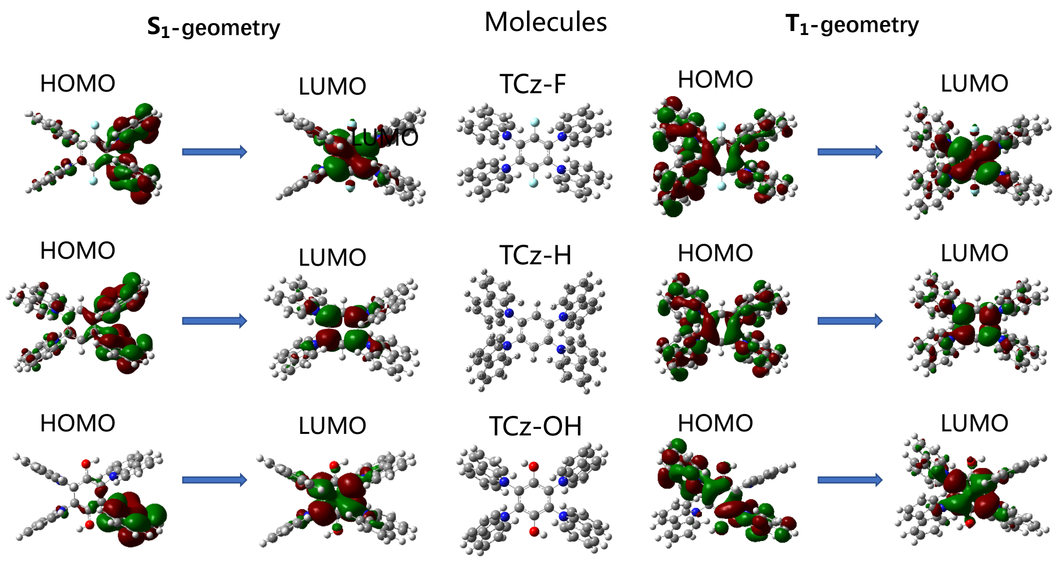

Supplementary Figure 34. Calculated transition orbitals at S_1 and T_1 state of TCz-F and TCz-H in THF solution.

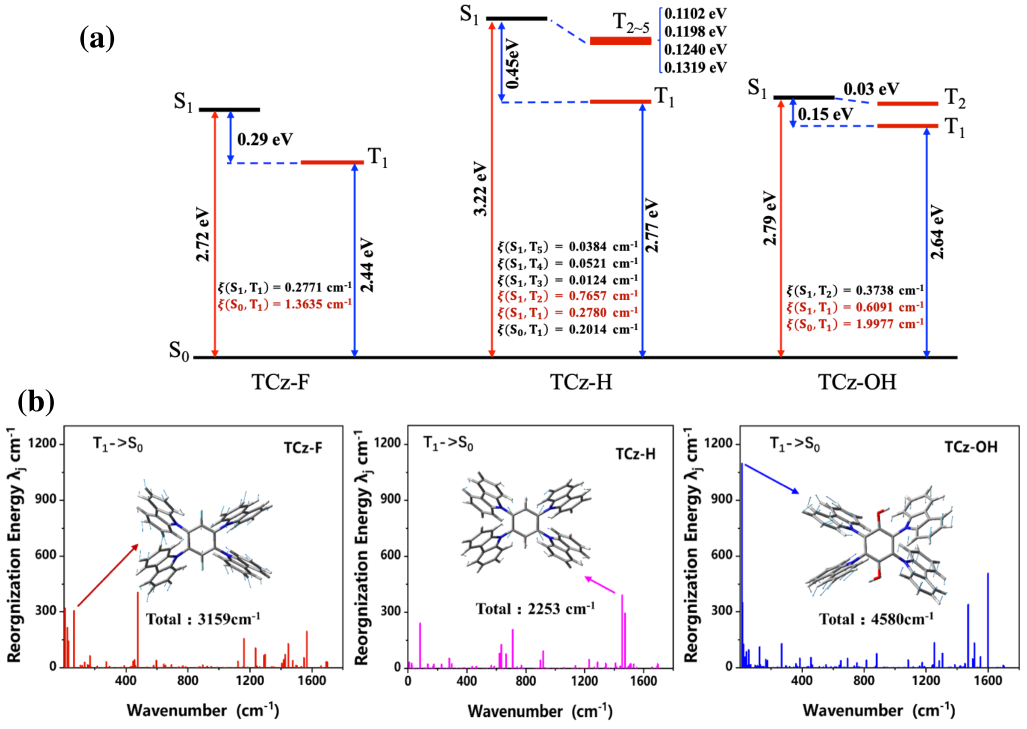

Supplementary Figure 35. (a) Calculated energy diagram and spin-orbital coupling (ξ) at S_1-geometry; (b) normal mode reorganization energy of TCz-F, TCz-H and TCz-OH in THF solution.

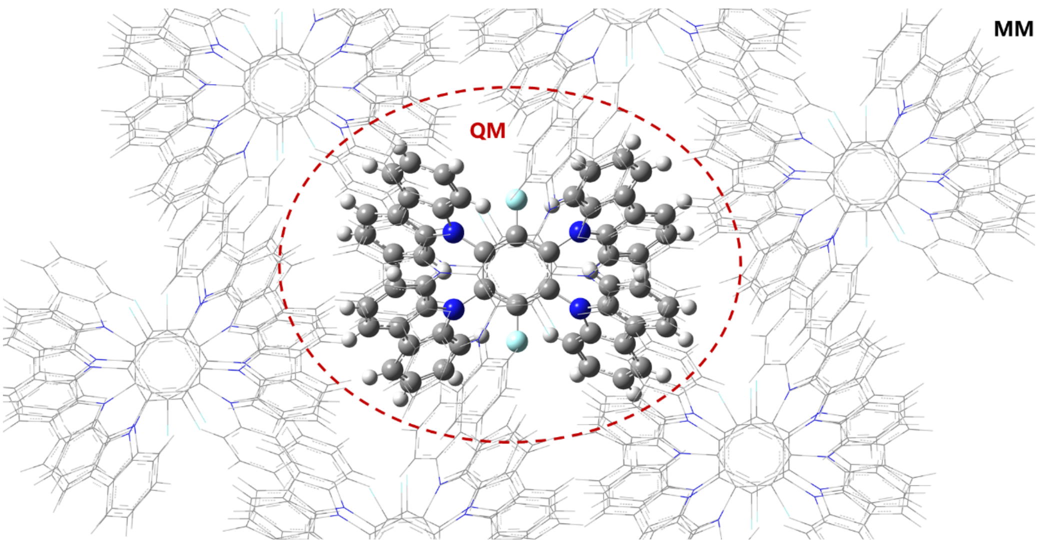

Supplementary Figure 36. Computational QM/MM model for FCz-F in the solid phase.

Supplementary Tables

Supplementary Table 1. Single crystal data of TCz-F-Cm, TCz-H-Cm and TCz-OH-Cm

	TCz-F	TCz-H	TCz-OH
Crystal system	orthorhombic	triclinic	triclinic
Space group	Pbcn	P-1	P-1
a/Å	13.828(3)	8.8851(5)	10.6840(15)
b/Å	18.330(4)	12.0609(7)	11.4759(16)
c/Å	17.405(4)	12.6893(10)	12.3938(17)
α/°	90	111.010(6)	108.446(3)
β/°	90	107.944(6)	111.810(3)
γ/°	90	98.675(5)	103.111(3)
Volume/$Å^3$	4411.2(17)	1154.21(14)	1541.4(3)
Z	4	1	1
$\rho_{calc}g/cm^3$	1.167	1.270	1.039
μ/mm^{-1}	0.075	0.598	0.064
F(000)	1608.0	466.0	402.0

Supplementary Table 2. Lifetime of TCz-F-Cm, TCz-H-Cm and TCz-OH-Cm in THF and water mixtures

f_w **(vol %)**	**0**	**30**	**50**	**60**	**70**	**80**	**90**	**95**
**TCz-F/ns**	7.31	9.36	10.81	11.44	7.93	8.47	7.39	7.68
**TCz-H/ns**	5.69	6.38	6.76	6.84	7.02	5.88	5.70	5.76
**TCz-OH/ns**	1.48	3.04	3.07	2.66	2.43	1.90	1.74	1.10

Supplementary Table 3. Temperature-dependent lifetime of TCz-F-Cm, TCz-H-Cm and TCz-OH-Cm in the solid state

	50 K	100K	150 K	200 K	250 K	300 K	350 K	400 K
**TCz-F/ms**	727.0	708.5	700.7	678.9	660.9	614.1	486.4	267.9
**TCz-H/ms**	128.0	123.6	118.2	87.5	31.7	23.8	19.6	10.7
**TCz-OH/ns**	11.1	11.3	11.1	12.2	12.1	12.4	12.3	11.8

Supplementary Table 4. EL performance of Device I-III

	J (mA cm^{-2})[a]	V (V)[a]	L (cd/m^2)[a]	η_C (cd/A)[a]	η_P (lm/W)[a]	η_{ext} (%)[a]	CIE (x, y)[a]
**Device I**	0.3	4.6	1.5	0.46	0.31	0.31	(0.282, 0.236)
	1	5.2	5.6	0.54	0.33	0.33	(0.307, 0.264)
	3.6	6.2	19.5	0.55	0.28	0.29	(0.357, 0.317)
	10	7.8	34.6	0.33	0.13	0.15	(0.424, 0.387)
	100	11.6	83.4	0.08	0.02	0.03	(0.451, 0.431)
**Device II**	0.3	4.8	1.5	0.52	0.34	0.47	(0.226, 0.179)
	1	5.4	4.7	0.48	0.28	0.42	(0.233, 0.186)
	6.3	7.6	20.2	0.32	0.13	0.18	(0.338, 0.307)
	10	8.4	23.9	0.25	0.09	0.13	(0.370, 0.342)
	100	12.8	64.1	0.06	0.02	0.03	(0.440, 0.431)
**Device III**	0.2	4.6	1.2	0.54	0.37	0.70	(0.179, 0.125)
	1	5.4	5.9	0.51	0.29	0.61	(0.188, 0.136)
	10	8.4	24.9	0.26	0.10	0.16	(0.307, 0.284)
	16.8	9.6	33.1	0.20	0.06	0.11	(0.347, 0.327)
	100	14	71.5	0.07	0.02	0.04	(0.432, 0.426)

Abbreviations: [a] The current density (*J*), luminescence (*L*), current efficiency (η_c), power efficiency (η_P), external quantum efficiency (η_{ext}) and Commission Internationale de L'Eclairage (CIE) coordinates.

Supplementary Table 5. Transient EL decay data for Device I at different current densities.

**J (mA cm^{-2})**	**R^2**	**<τ> (μs)**	**τ_1 (μs)**	**τ_2 (μs)**	**A_1**	**A_2**
**1**	0.954	1.17	0.57	14.78	138485	235
**3.6**	0.974	3.43	0.95	15.86	25305	302
**10**	0.984	9.83	1.91	21.19	4004	252

[a] The transient EL decay data was fitted by double-exponential function and the average fluorescence lifetimes (<τ>) were calculated by $<\tau> = \Sigma A_i\tau_i^2/\Sigma A_i\tau_i$, where A_i is the pre-exponential for lifetime τ_i.

Supplementary Table 6. Electroluminescence Performance of the OLEDs

	λ_{EL} (nm)	V_{on} (V)	L (cd/m^2)	η_C (cd/A)	η_P (lm/W)	EQE (%)	CIE (x, y)
**IV**	442	5.0	63.41	0.82	0.51	1.06	(0.155, 0.101)

Abbreviations: [a)] V_{on} = turn-on voltage at 1cd/m^2; [b)] V = voltage; η_C = current efficiency; η_P = power efficiency; η_{ext} = external quantum efficiency; L = luminance; [c)] CIE = Commission International de I'Eclairage coordinates at 1 mA/cm^2; [d)] λ_{EL} = maxima of electroluminescent spectra at 1 mA/cm^2.

Supplementary Table 7. Electroluminescence Performance of Device V-VIII

	λ_{EL} (nm)	V_{on} (V)	L (cd/m^2)	η_C (cd/A)	η_P (lm/W)	EQE (%)	CIE (x, y)
**V**	442	4.1	163.8	0.61	0.45	0.70	(0.156, 0.117)
**VI**	436	4.1	382.4	0.56	0.40	0.63	(0.158, 0.121)
**VII**	436	4.0	380.6	0.57	0.39	0.65	(0.158, 0.122)
**VIII**	436	4.0	401.6	0.57	0.42	0.65	(0.158, 0.121)

Abbreviations: [a)] V_{on} = turn-on voltage at 1cd/m^2; [b)] V = voltage; η_C = current efficiency; η_P = power efficiency; η_{ext} = external quantum efficiency; L = luminance; [c)] CIE = Commission International de I'Eclairage coordinates at 1 mA/cm^2; [d)] λ_{EL} = maxima of electroluminescent spectra at 1 mA/cm^2.

Supplementary Table 8. Rate constants of TCz-F, TCz-H and TCz-OH.

**Emitter**	k_r^{Fluo} (s^{-1})	k_{nr}^{Fluo} (s^{-1})	k_{ISC} (s^{-1})	k_r^{Phos} (s^{-1})	k_{nr}^{Phos} (s^{-1})
**TCz-F**	4.03×10^7	8.76×10^7	1.01×10^7	0.12	1.51
**TCz-H**	2.99×10^6	1.65×10^8	8.08×10^6	1.93	40.08
**TCz-OH**	3.15×10^6	7.75×10^7	----	----	-----

Supplementary Table 9. Calculated vertical excitation energy (eV) at the optimized geometry in the ground state (corresponding to the absorption spectral peak position, nm), and the oscillator strength (*f*), taking TCz-F in THF solution an example. The orbital properties are only given for the electric dipole-allowed transition.

	ΔE_v	*f*	Transition	Orbital property
S_1	3.50/354	0.1936	H → L	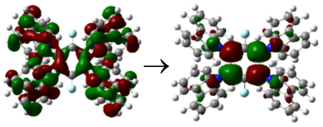 CT
S_2	3.64/341	0.3715	H-1→ L	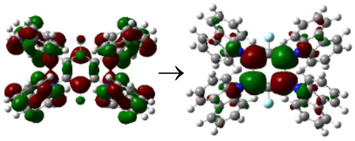 CT
S_3	3.75/331	0.0000	H-2 → L	-
S_4	3.81/325	0.0000	H-3 → L	-
S_5	4.01/309	0.0069	H-4 → L	-
S_6	4.03/308	0.0000	H-5 → L	-
S_7	4.09/303	0.0045	H-6 → L	-
S_8	4.09/303	0.0000	H-7 → L	-
S_9	4.16/298	0.2724	H → L+2	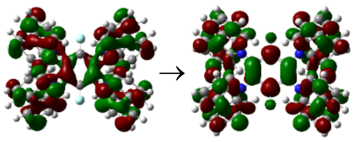 LE
S_{10}	4.24/293	0.0000	H → L+1	-

Supplementary Notes

PL quantum yield (Φ_{PL}), PL lifetime (τ), rate constant of radiative decay (k_r), and non-radiative decay (k_{nr}) are defined as below:

Supplementary Note 1: Fluorescence Radiative Rate Constants

$$k_r^{Fluo} = {}^{\emptyset_{Fluo}}\!/_{\tau_{Fluo}}$$

Supplementary Note 2: Fluorescence Non-radiative Rate Constants

$$k_{nr}^{Fluo} = {}^{(1 - \emptyset_{Fluo} - \emptyset_{Phos})}\!/_{\tau_{Fluo}}$$

Supplementary Note 3: ISC Rate Constants

$$k_{ISC} = {}^{\emptyset_{Phos}}\!/_{\tau_{Fluo}}$$

Supplementary Note 4: Phosphorescence Non-radiative Rate Constants

$$k_r^{Phos} = {}^{\emptyset_{Phos}}\!/_{\tau_{Phos}}$$

Supplementary Note 5: Phosphorescence Non-radiative Rate Constants

$$k_{nr}^{Phos} = {}^{(1 - \emptyset_{Phos})}\!/_{\tau_{Phos}}$$
